# Supplementary material for: Strategies found not to be suitable for stabilizing high steroid hydroxylation activities of CYP450 BM3-based whole-cell biocatalysts
Source: PLoS One. 2024 Sep 6;19(9):e0309965. doi: 10.1371/journal.pone.0309965 (PMC11379211; doi:10.1371/journal.pone.0309965)
Supplement: S1 Table — (PDF) [file pone.0309965.s001.pdf]

S1 Table. Primer sequences and PCR templates used for the construction of the plasmids used in this study.

Plasmid	Primers	Sequence (5’→3’)[2]	PCR template[1]	Additional information[3]
palk-ksa14m	PCK0017_for<br>PCK0018_rev	ATTTAATAAAAATT**GGAGAA**TTCCATATGGCAATTAAAGAAATGCCTC<br>ATCTTCTCTCATCCGCCAAAACAGAAGCTTCGATCGTTACCCAGCCCACACGTC	*ksa14m* gene synthesis product	insertion of *ksa14m* PCR product into pCOM10 (cut with NdeI and HindIII)
palk-ksa14m-alkL	PCK0019_for<br>PCK0020_rev	AAAGACGTGTGGGCTGGGTAACGATCG**TAACTTTAAGAAGGAGATATACC**TACTAGATGAGTTTTTCTAATTATAAAGTAATCG<br>CATCCGCCAAAACAGAAGCTTCGATCGTTAGAAAACATATGACGCACC	pCW3A4LS (derived from pLAFR3-AlkL (Cornelissen *et al.*, 2013))	insertion of *alkL* PCR product into palk-ksa14m (cut with PvuI)
plac-ksa14m	PCK0013_for<br>PCK0014_rev	TAGGAACCAGTACT**GGAGAA**TTCCATATGGCAATTAAAGAAATGCCTC<br>ATCTTCTCTCATCCGCCAAAACAGAAGCTTACTAGTTTACCCAGCCCACACGTC	*ksa14m* gene synthesis product	insertion of *ksa14m* PCR product into pCOM10_1A1_lac (cut with NdeI and HindIII)
plac-ksa14m-alkL	PCK0015_for<br>PCK0016_rev	GCAAAAGACGTGTGGGCTGGGTAAACTAGT**TAACTTTAAGAAGGAGATATACC**TACTAGATGAGTTTTTCTAATTATAAAGTAATCG<br>TCTCATCCGCCAAAACAGAAGCTTACTAGTTTAGAAAACATATGACGCACC	pCW3A4LS (derived from pLAFR3-AlkL (Cornelissen *et al.*, 2013))	insertion of *alkL* PCR product into plac-ksa14m (cut with BcuI)
ptac-ksa14m	PCK0021_for<br>PCK0018_rev	TGACCAACAGTACT**GGAGAA**TTCCATATGGCAATTAAAGAAATGCCTC<br>ATCTTCTCTCATCCGCCAAAACAGAAGCTTCGATCGTTACCCAGCCCACACGTC	*ksa14m* gene synthesis product	insertion of *ksa14m* PCR product into pCOM10_tac (cut with NdeI and HindIII)
ptac-ksa14m-alkL	PCK0019_for<br>PCK0020_rev	AAAGACGTGTGGGCTGGGTAACGATCG**TAACTTTAAGAAGGAGATATACC**TACTAGATGAGTTTTTCTAATTATAAAGTAATCG<br>CATCCGCCAAAACAGAAGCTTCGATCGTTAGAAAACATATGACGCACC	pCW3A4LS (derived from pLAFR3-AlkL (Cornelissen *et al.*, 2013))	insertion of *alkL* PCR product into ptac-ksa14m (cut with PvuI)
plac-ksa14m-fhuAΔ1-160	PCK0082_for<br>PCK0083_rev	GCAAAAGACGTGTGGGCTGGGTAAACTAGT**TAACTTTAAGAAGGAGATATACC**TACTAGATGGCGCGTTCCAAAAC<br>TCTCATCCGCCAAAACAGAAGCTTACTAGTTTAGAAACGGAAGGTTGC	pETM11_KSA14m_fhuAΔ1-160 (Bertelmann *et al.*, 2022)	insertion of *fhuAΔ1-160* PCR product into plac-ksa14m-alkL (cut with BcuI)
plac-ksa14m-todX	PCK0084_for<br>PCK0085_rev	GCAAAAGACGTGTGGGCTGGGTAAACTAGT**TAACTTTAAGAAGGAGATATACC**TACTAGATGAAGATTGCCAGCGTG<br>TCTCATCCGCCAAAACAGAAGCTTACTAGTTTAAAAATTTTTGCTATAGGAAACC	pETM11_KSA14m_todX (Bertelmann *et al.*, 2022)	insertion of *todX* PCR product into plac-ksa14m-alkL (cut with BcuI)
pET154	PCK0075_for<br>PCK0076_rev	ATTGTGAGCGGATAACAATTCCCCTCTAGAAATAATTTTGTT**TAAC**<br>GGTGGTGGTGGTGGTGCTCGAGTGCGGCCGCTTAAAGCTTGTCGACGG	*cyp154c5* gene synthesis product	insertion of *cyp154c5* PCR product into pETM11 (cut with XbaI and NotI)
pET154-camAB	PCK0027_for (camA)<br>PCK0028_rev (camA)<br>PCK0029_for (camB)<br>PCK34_rev (camB)	TGGGGCGCTAAGAGCTCCGTCGACAAGCTT**TAACTTTAAGAAGGAGATATACC**ATGAACGCAAACGACAACGTGGTC<br>CTCCTTCTTAAAGTTACTCTAGTAGGATCCCTCTAGTATTAGGCACTACTCAGTTCAGCTTTG<br>CTACTAGAG**TAACTTTAAGAAGGAGATATACC**ATGTCTAAAGTAGTGTATGTGTCAC<br>GGTGCTCGAGTGCGGCCGCAAGCTAAGCTTTTACCATTGCCTATCGGGAAC	genomic DNA of *Pseudomonas putida* DSM50198 (DSMZ – German Collection of Microorganisms and Cell Cultures GmbH)	simultaneous insertion of *camA* and *camB* PCR products into pET154 (cut with HindIII)
pET154-camAB-alkL	PCK0035_for<br>PCK0036_rev	AGGCAATGGTAAAAGCTTAGCTTGCGGCCGC**TAACTTTAAGAAGGAGATATACC**ATGAGTTTTTCTAATTATAAAGTAATCG<br>GGTGGTGGTGGTGGTGCTCGAGTGCGGCCGCTTAGAAAACATATGACGCACC	pCW3A4LS (derived from pLAFR3-AlkL (Cornelissen *et al.*, 2013))	insertion of *alkL* PCR product into pET154-camAB (cut with NotI)

pET154A	PCK0078_for<br>PCK0079_rev	AGAGCTCCGTCGACAAGCTTTAAGCGGCCGC**TAACTTTAAGAAGGAGATATACC**ATGAGTTTTTCTAATTATAAAGTAATC<br>GGTGGTGGTGGTGGTGCTCGAGTGCGGCCGCTTAGAAAACATATGACGCACC	pCW3A4LS (derived from pLAFR3-AlkL (Cornelissen *et al.*, 2013))	insertion of *alkL* PCR product into pET154
pET106	PCK0075_for<br>PCK0077_rev	ATTGTGAGCGGATAACAATTCCCCTCTAGAAATAATTTTGTT**TAAC**<br>GGTGGTGGTGGTGGTGCTCGAGTGCGGCCGCTTAAAGCTTGTCGACGGAG	*cyp106a2* gene synthesis product	insertion of *cyp106a2* PCR product into pETM11
pET106A	PCK0078_for<br>PCK0079_rev	AGAGCTCCGTCGACAAGCTTTAAGCGGCCGC**TAACTTTAAGAAGGAGATATACC**ATGAGTTTTTCTAATTATAAAGTAATC<br>GGTGGTGGTGGTGGTGCTCGAGTGCGGCCGCTTAGAAAACATATGACGCACC	pCW3A4LS (derived from pLAFR3-AlkL (Cornelissen *et al.*, 2013))	insertion of *alkL* PCR product into pET106
ptac154	PCK0063_for<br>PCK0064_rev	AGTCCGTTTAGGTGTTTTCACGAGCAATTGCCTCTAGAAATAATTTTGTT**TAAC**<br>TGGCTGCAGGTCGACGGATCCCGGGCGCGCCTTAAAGCTTGTCGACGG	*cyp154c5* gene synthesis product	insertion of *cyp154c5* PCR product in pCOM10_tac (cut with MunI and SgsI)
ptac154A	PCK0065_for<br>PCK0066_rev	AGAGCTCCGTCGACAAGCTTTAAGGCGCGCC**TAACTTTAAGAAGGAGATATACC**ATGAGTTTTTCTAATTATAAAGTAATC<br>ACAGAAGCTTGGCTGCAGGTCGACGGATCCTTAGAAAACATATGACGCACC	pCW3A4LS (derived from pLAFR3-AlkL (Cornelissen *et al.*, 2013))	insertion of *alkL* PCR product in ptac154 (cut with SgsI and BamHI)
ptac106	PCK0067_for<br>PCK0068_rev	AGTCCGTTTAGGTGTTTTCACGAGCAATTGCCTCTAGAAA**TAATTTTGTTTAACTTTAAGAAGGAGATATACC**ATGAAGGAGGTGATCGCAG<br>TGGCTGCAGGTCGACGGATCCCGGGCGCGCCTTAAAGCTTGTCGACGGAGCTCTTACATACGACTCGCTTTCAG	*cyp106a2* gene synthesis product	insertion of *cyp106a2* PCR product in pCOM10_tac (cut with MunI and SgsI)
ptac106A	PCK0065_for<br>PCK0066_rev	AGAGCTCCGTCGACAAGCTTTAAGGCGCGCC**TAACTTTAAGAAGGAGATATACC**ATGAGTTTTTCTAATTATAAAGTAATC<br>ACAGAAGCTTGGCTGCAGGTCGACGGATCCTTAGAAAACATATGACGCACC	pCW3A4LS (derived from pLAFR3-AlkL (Cornelissen *et al.*, 2013))	insertion of *alkL* PCR product in ptac106 (cut with SgsI and BamHI)
pACYC-camA	PCK0059_for<br>PCK0060_rev	GTTTAACTTTAAT**AAGGAG**ATATACCCATGAACGCAAACGACAACGTGGTC<br>TGTCGACCTGCAGGCGCGCCGAGCTCTCAGGCACTACTCAGTTCAGCTTTG	genomic DNA of *Pseudomonas putida* DSM50198 (DSMZ – German Collection of Microorganisms and Cell Cultures GmbH)	insertion of *camA* PCR product into pACYC_DuetI (cut with NcoI and SacI)
pACYC-camAB	PCK0061_for<br>PCK0062_rev	GTTAAGTATAAG**AAGGAG**ATATACATATGTCTAAAGTAGTGTATGTGTCAC<br>GCGATCGCGTGGCCGGCCGATATCCAATTGTTACCATTGCCTATCGGGAAC	genomic DNA of *Pseudomonas putida* DSM50198 (DSMZ – German Collection of Microorganisms and Cell Cultures GmbH)	insertion of *camB* PCR product into pACYC-camA (cut with NdeI and MunI)

[1] Genes encoding the respective CYP450s, redox partner proteins, or outer membrane proteins were amplified from various sources using suitable primers.
[2] Primers contained (parts of) ribosomal binding sites (**bold**), restriction sites, and 25 bp overhangs complementary to the plasmid backbone.
[3] The resulting PCR products were inserted into the different vectors by *in vitro assembly* (Gibson *et al.*, 2009). Final plasmid constructs were then introduced into the desired host organism.

References

Bertelmann, C., Mock, M., Koch, R., Schmid, A., and Bühler, B. (2022) Hydrophobic outer membrane pores boost testosterone hydroxylation by cytochrome P450 monooxygenase BM3 containing cells. *Front Catal* **2**: 887458.

Cornelissen, S., Julsing, M.K., Volmer, J., Riechert, O., Schmid, A., and Bühler, B. (2013) Whole-cell-based CYP153A6-catalyzed (*S*)-limonene hydroxylation efficiency depends on host background and profits from monoterpene uptake via AlkL. *Biotechnol Bioeng* **110**: 1282-1292.

Gibson, D.G., Young, L., Chuang, R.-Y., Venter, J.C., Hutchison, C.A., 3rd, and Smith, H.O. (2009) Enzymatic assembly of DNA molecules up to several hundred kilobases. *Nat Methods* **6**: 343-345.
