# Supplementary material for: Strategies found not to be suitable for stabilizing high steroid hydroxylation activities of CYP450 BM3-based whole-cell biocatalysts
Source: PLoS One. 2024 Sep 6;19(9):e0309965. doi: 10.1371/journal.pone.0309965 (PMC11379211; doi:10.1371/journal.pone.0309965)
Supplement: S2 Table — (PDF) [file pone.0309965.s002.pdf]

S2 Table. Components and settings of site-directed mutagenesis PCR to introduce M82A and L188P amino acid exchanges into KSA14m.

**Composition of PCR mixture**		**Temperature profile**			
**PCR component**	**Final concentration**	**Step**	**M82A**	**L188P**	
5X Q5 Reaction Buffer	1X	Initial denaturation	98°C, 90 sec	98°C, 90 sec	
10 mM dNTPS	200 µM	Denaturation	98°C, 30 sec	98°C, 30 sec	35 cycles!
10 µM Forward primer	0.5 µM	Annealing	59°C, 30 sec	68°C, 30 sec	
10 µM Reverse primer	0.5 µM	Elongation	72°C, 4 min 35 sec	72°C, 4 min 36 sec	
Template DNA pETM11-ksa14m-alkL	20 ng	Final elongation	72°C, 10 min	72°C, 10 min	
Q5 High Fidelity Polymerase	1 U	Hold	4°C	4°C	

**Plasmid**	**Primer**	**Sequence (5'→3')**
pETM11-ksa14m(M82A)-alkL	M82A_for	TCGTGATTTTGCAGGAGACGGG
	M82A_rev	AGAAATTTAAGCGCTTGAC
pETM11-ksa14m(L188P)-alkL	L188P_for	ATGAACAAGCCACAGCGAGCAAATC
	L188P_rev	GTTCATTGCTTCATCCAGTGCAC
