# Supplementary material for: Strategies found not to be suitable for stabilizing high steroid hydroxylation activities of CYP450 BM3-based whole-cell biocatalysts
Source: PLoS One. 2024 Sep 6;19(9):e0309965. doi: 10.1371/journal.pone.0309965 (PMC11379211; doi:10.1371/journal.pone.0309965)
Supplement: S3 Table — (PDF) [file pone.0309965.s003.pdf]

S3 Table. Composition and preparation of minimal media used in this study.

**M9 medium** (Sambrook and Russell, 2001)		**M9* medium** (Panke *et al.*, 1999)		**Riesenberg (RB) medium** (Riesenberg *et al.*, 1991)	
**Component**	**Concentration**	**Component**	**Concentration**	**Component**	**Concentration**
$Na_2HPO_4 \cdot 2H_2O$	8.5 g L^{-1}	$Na_2HPO_4 \cdot 2H_2O$	25.5 g L^{-1}	$(NH_4)_2HPO_4$	13.3 g L^{-1}
KH_2PO_4	3.0 g L^{-1}	KH_2PO_4	9.0 g L^{-1}	KH_2PO_4	4.0 g L^{-1}
NaCl	0.5 g L^{-1}	NaCl	0.5 g L^{-1}	Citric acid	1.7 g L^{-1}
NH_4Cl	1.0 g L^{-1}	NH_4Cl	1.0 g L^{-1}	-	-
Main medium components were dissolved in MilliQ water and pH was adjusted to 7.2 using NaOH (10 M). After sterilization, the medium was supplemented with the following ingredients:		Main medium components were dissolved in MilliQ water and pH was adjusted to 7.2 using NaOH (10 M). After sterilization, the medium was supplemented with the following ingredients:		Main medium components were dissolved in MilliQ water and pH was adjusted to 6.8 using 25% ammonia and then further increased to 7.2 by 10 M NaOH. After sterilization, the medium was supplemented with the following ingredients:	
**Supplement**	**Concentration**	**Supplement**	**Concentration**	**Supplement**	**Concentration**
$MgSO_4$	2 mM	$MgSO_4$	2 mM	$MgSO_4$	2.5 mM
US* trace elements (Panke *et al.*, 1999)	0.1% (v/v)	US* trace elements (Panke *et al.*, 1999)	0.1% (v/v)	US* trace elements (Panke *et al.*, 1999)	0.1% (v/v)
D-glucose	0.5% (w/v)	D-glucose	0.5% (w/v)	D-glucose	0.5% (w/v)

References

Panke, S., Meyer, A., Huber, C.M., Witholt, B., and Wubbolts, M.G. (1999) An alkane-responsive expression system for the production of fine chemicals. *Appl Environ Microbiol* **65**: 2324-2332.

Riesenberg, D., Schulz, V., Knorre, W.A., Pohl, H.-D., Korz, D., Sanders, E.A.*, et al.* (1991) High cell density cultivation of *Escherichia coli* at controlled specific growth rate. *J Biotechnol* **20**: 17-27.

Sambrook, J., and Russell, D.W. (2001) *Molecular cloning: a laboratory manual*. Cold Spring Harbor, NY: Cold Spring Harbor Laboratory Press.
