# Supplementary figures and images for: Strategies found not to be suitable for stabilizing high steroid hydroxylation activities of CYP450 BM3-based whole-cell biocatalysts
Source: PLoS One. 2024 Sep 6;19(9):e0309965. doi: 10.1371/journal.pone.0309965 (PMC11379211; doi:10.1371/journal.pone.0309965)

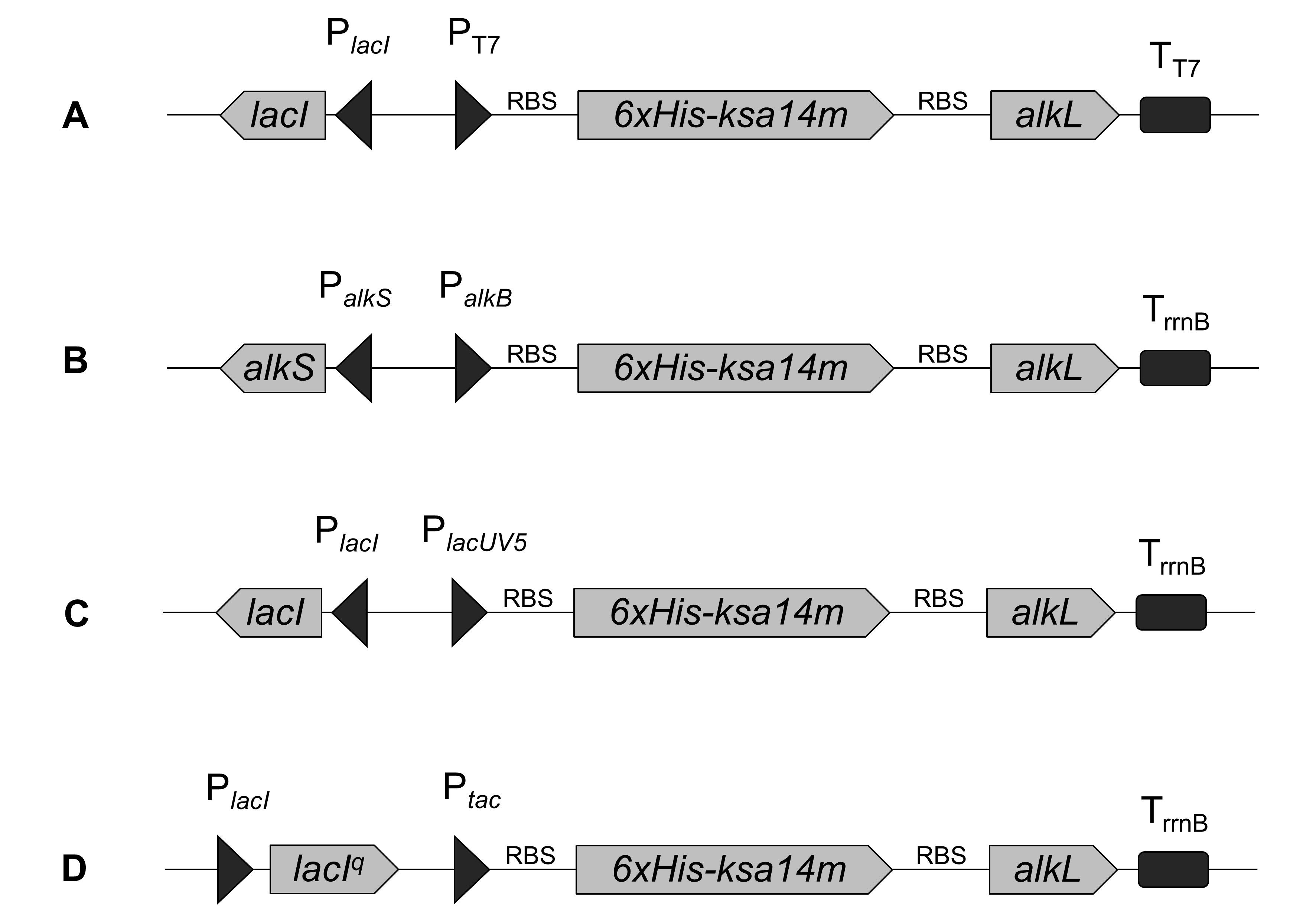

Supplement: S1 Fig — Heterologous gene expression was either realized via (A) the T7 promoter (PT7), (B) the alkB promoter (PalkB), (C) the lacUV5 promoter (PlacUV5), or (D) the tac promoter (Ptac) based on either LacIq (A, C, D) or AlkS (B) as regulators. The creation of plasmids pETM11-ksa14m-alkL (A), palk-ksa14m-alkL (B), plac-ksa14m-alkL (C), and ptac-ksa14m-alkL (D) is explained in S1 Table. (JPG) [file pone.0309965.s004.jpg]

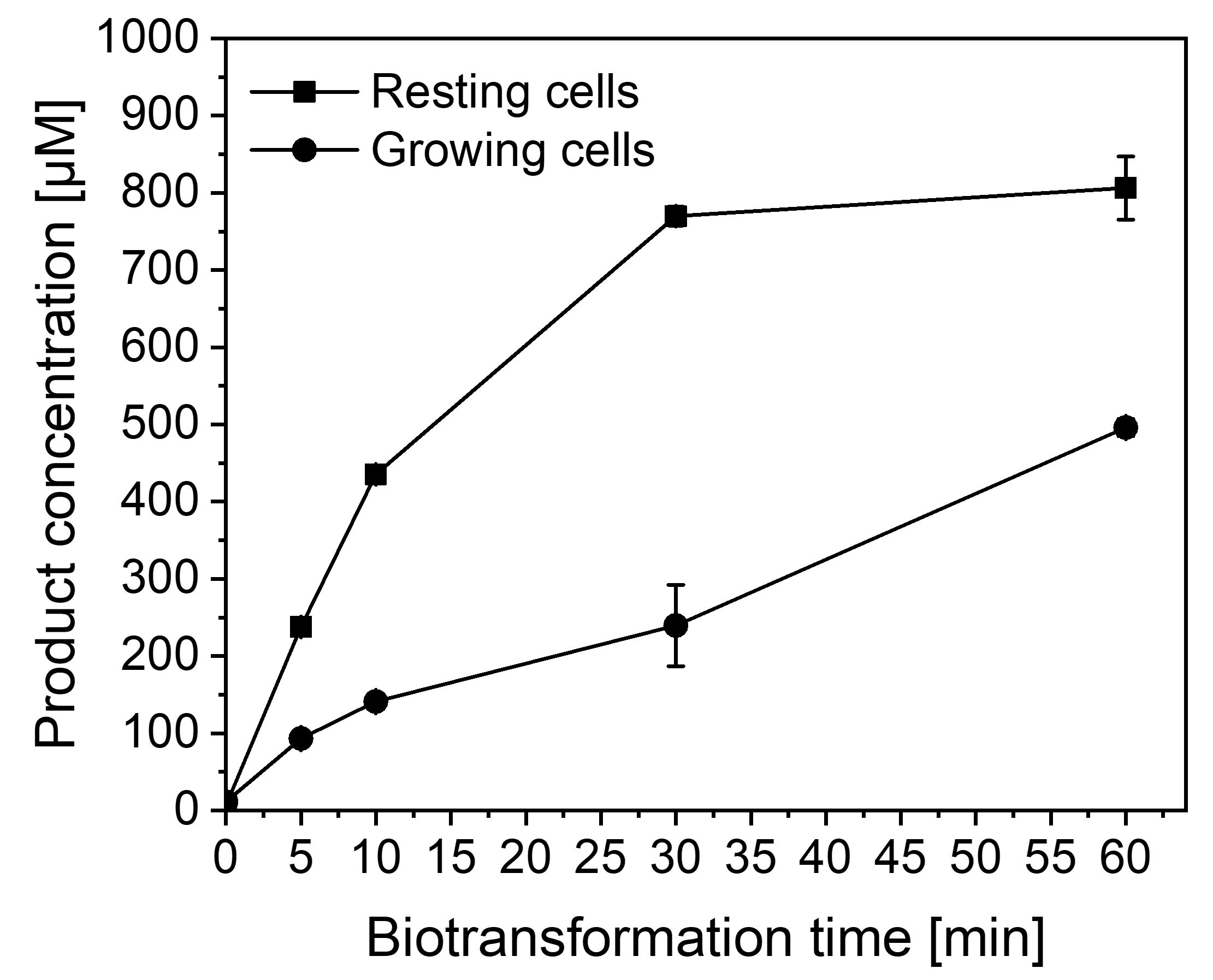

Supplement: S2 Fig — Microorganisms were cultivated in M9 medium containing 0.5% (w/v) glucose and heterologous gene expression was induced with 0.1 mM IPTG. Biotransformations were conducted as described in the Materials and Methods Section. Average values and standard deviations of two biological replicates are shown. (TIF) [file pone.0309965.s005.tif]

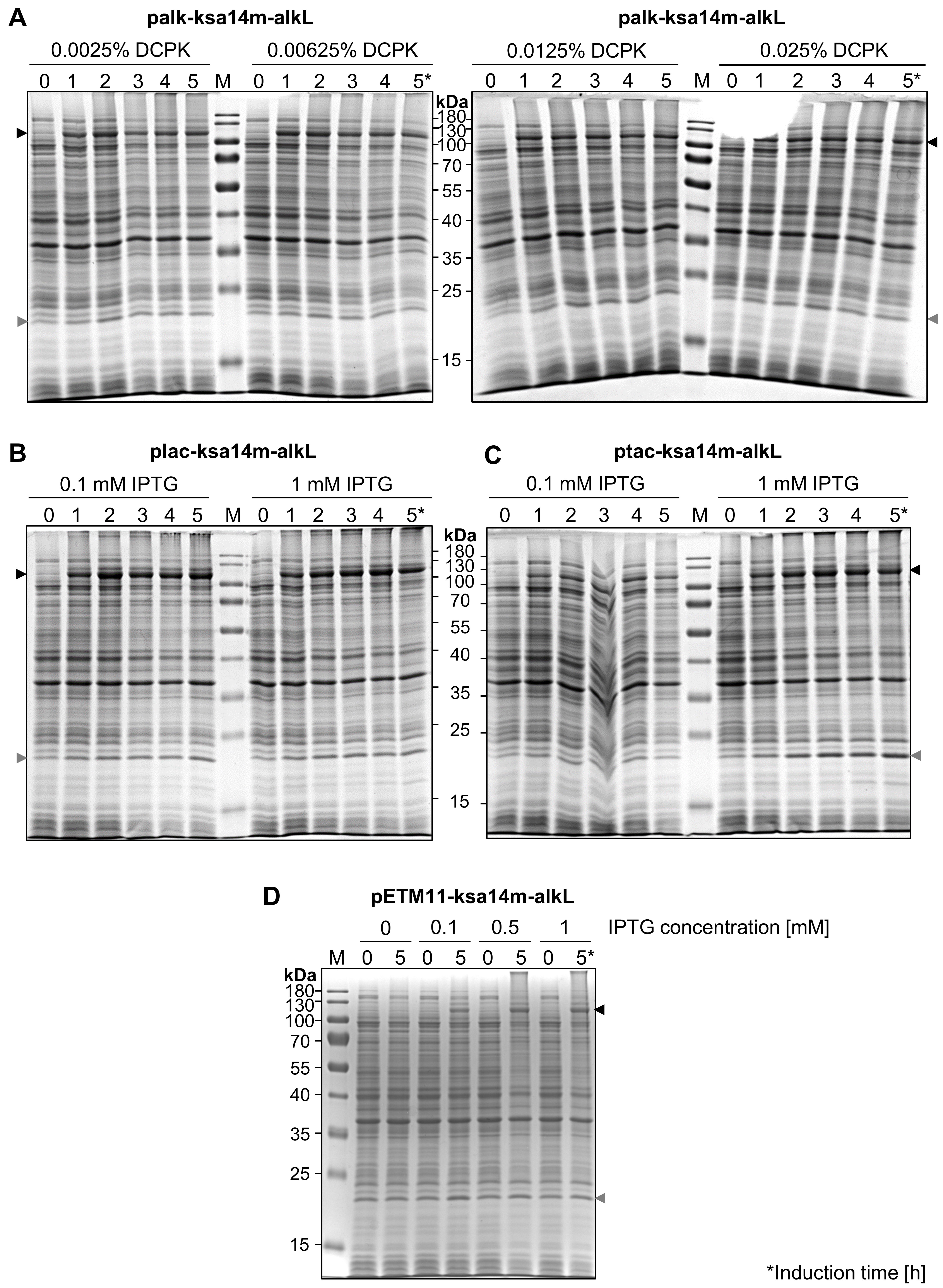

Supplement: S3 Fig — Microorganisms were cultivated in M9 medium containing 0.5% (w/v) glucose and heterologous gene expression was induced in the early exponential phase. (A) AlkS-PalkB-based regulatory system, induced with different DCPK concentrations. (B) LacI-PlacUV5-based expression system, induced with different IPTG concentrations. (C) LacIq-Ptac-based expression system, induced with different IPTG concentrations. (D) LacI-PT7-based regulatory system, either uninduced or induced with different IPTG concentrations. (JPG) [file pone.0309965.s006.jpg]

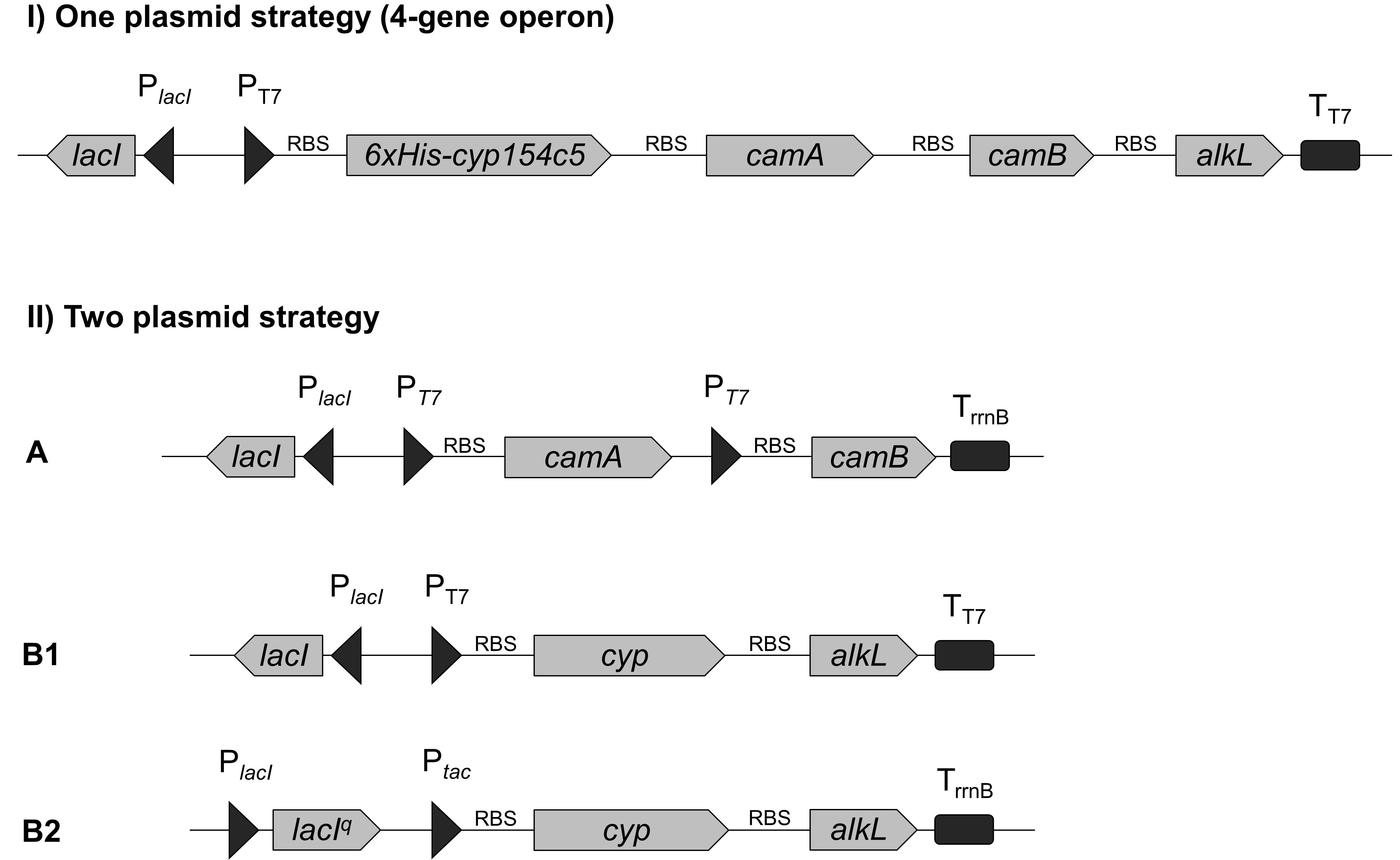

Supplement: S5 Fig — Necessary genes encoded either CYP154C5 or CYP106A2 (cyp), redox partner proteins (camA for putidaredoxin reductase, PDR, and camB for putidaredoxin, Pd), as well as the hydrophobic outer membrane protein AlkL (alkL). I) One plasmid strategy: one vector for the synthesis of 6xHis-tagged CYP154C5, PDR, Pd, and AlkL under control of the LacI-PT7-based expression system. II) Two plasmid strategy: cyp/alkL and camA/camB were expressed from two separate plasmids. Final bacterial strains always contained plasmid A (pACYC-camAB) carrying camA and camB under control of two separate T7 promoters. In addition, the cells harbored either plasmid B1 or B2 carrying the respective cyp genes and alkL under control of PT7 (B1) or Ptac (B2). Plasmid construction is detailed in S1 Table. (JPG) [file pone.0309965.s008.jpg]

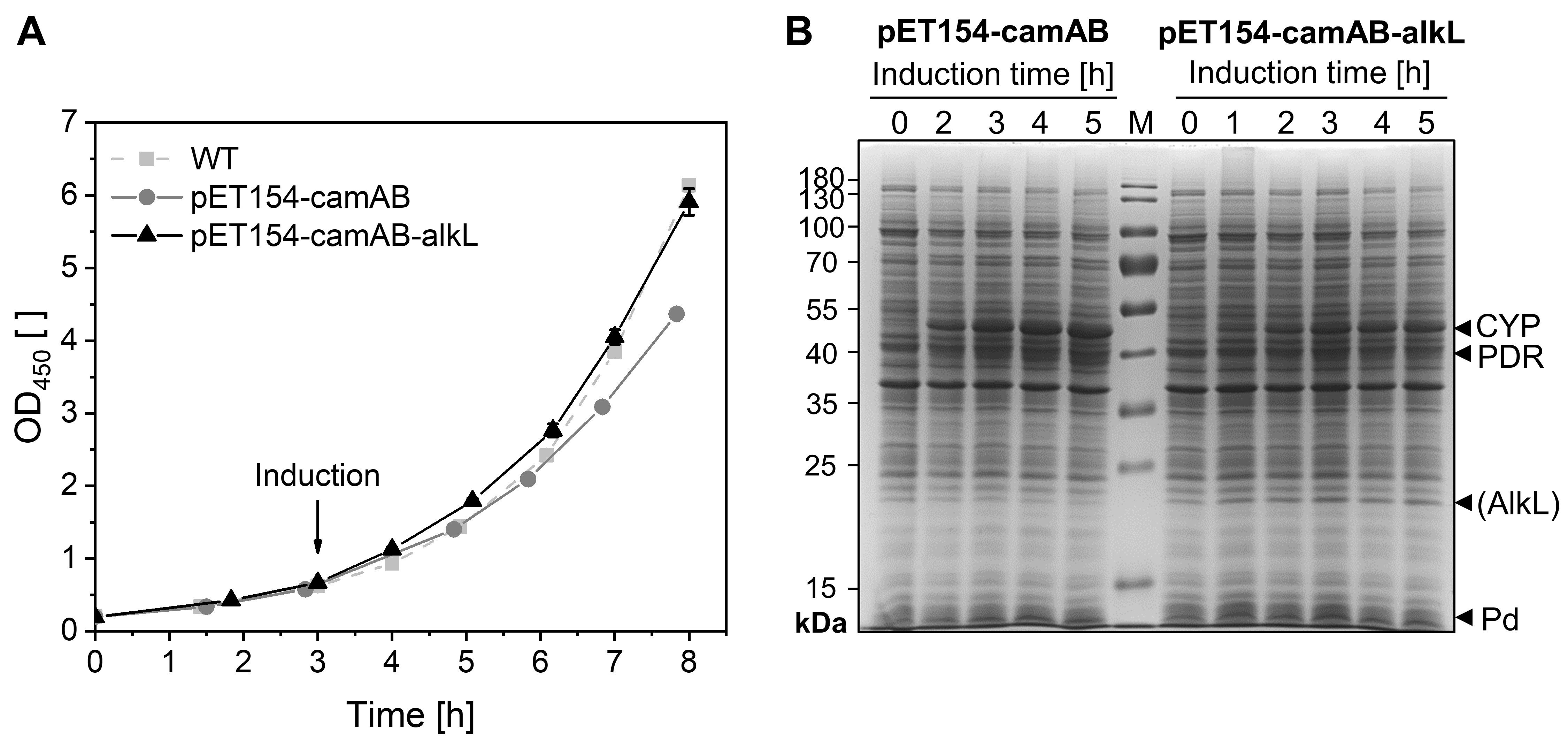

Supplement: S6 Fig — Microorganisms were cultivated in M9 medium containing 0.5% (w/v) glucose and heterologous gene expression was induced with 0.1 mM IPTG. (A) Growth of recombinant strains compared to the wild type (WT). Average values and standard deviations of biological duplicates are presented. (B) SDS-PAGE analysis of heterologous protein levels achieved with the respective strains after different times of induction. Expected proteins are marked with arrows (CYP154C5, 45 kDa; PDR, 45 kDa; AlkL, 23 kDa; Pd, 11 kDa). (JPG) [file pone.0309965.s009.jpg]

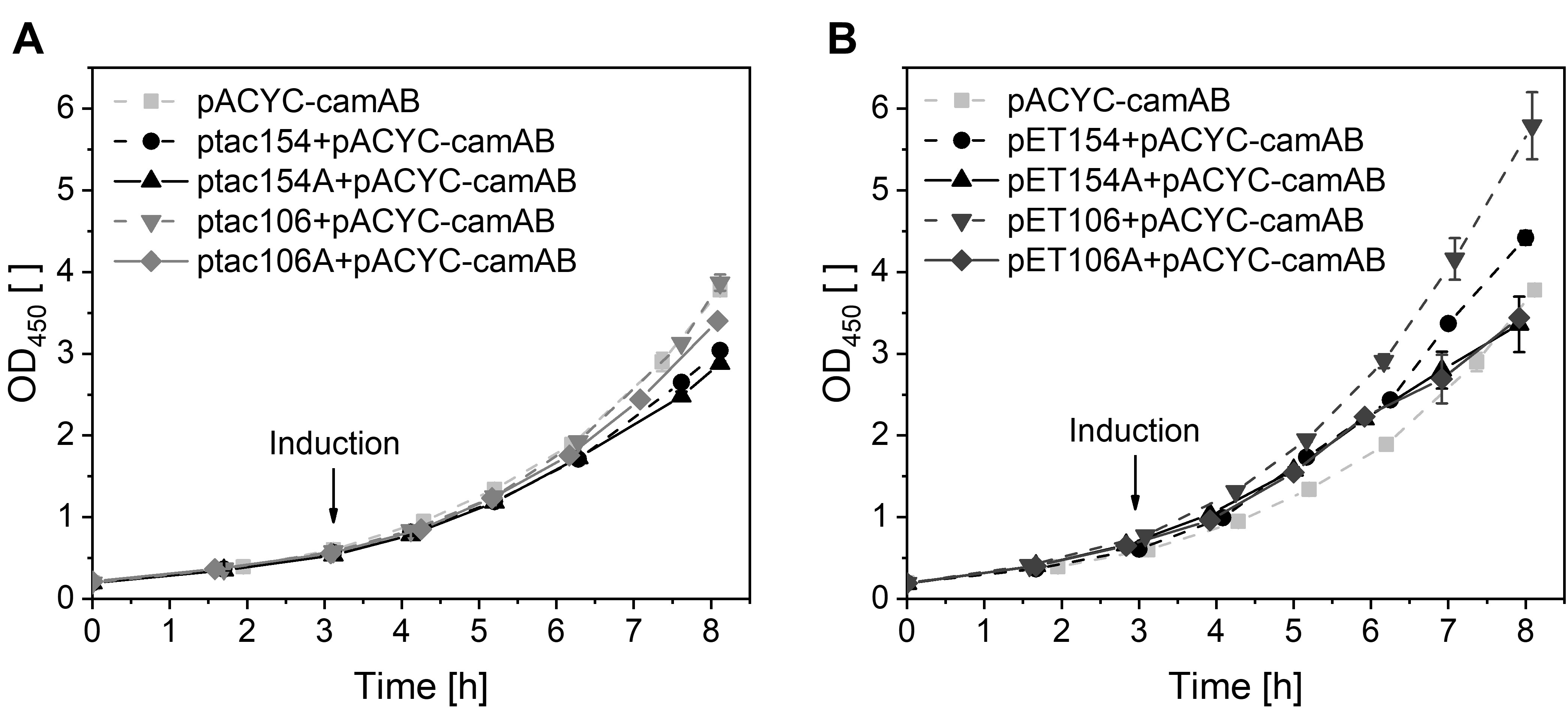

Supplement: S8 Fig — All strains contained pACYC-camAB and a second plasmid carrying cyp154c5 or cyp106a2 together with or without alkL on pCOM10_tac (A) or pETM11 (B). Recombinant bacteria were cultivated in M9 medium supplemented with 0.5% (w/v) glucose and heterologous gene expression was induced with 0.1 mM IPTG. Data points represent average values and standard deviations of two biological replicates. (JPG) [file pone.0309965.s011.jpg]

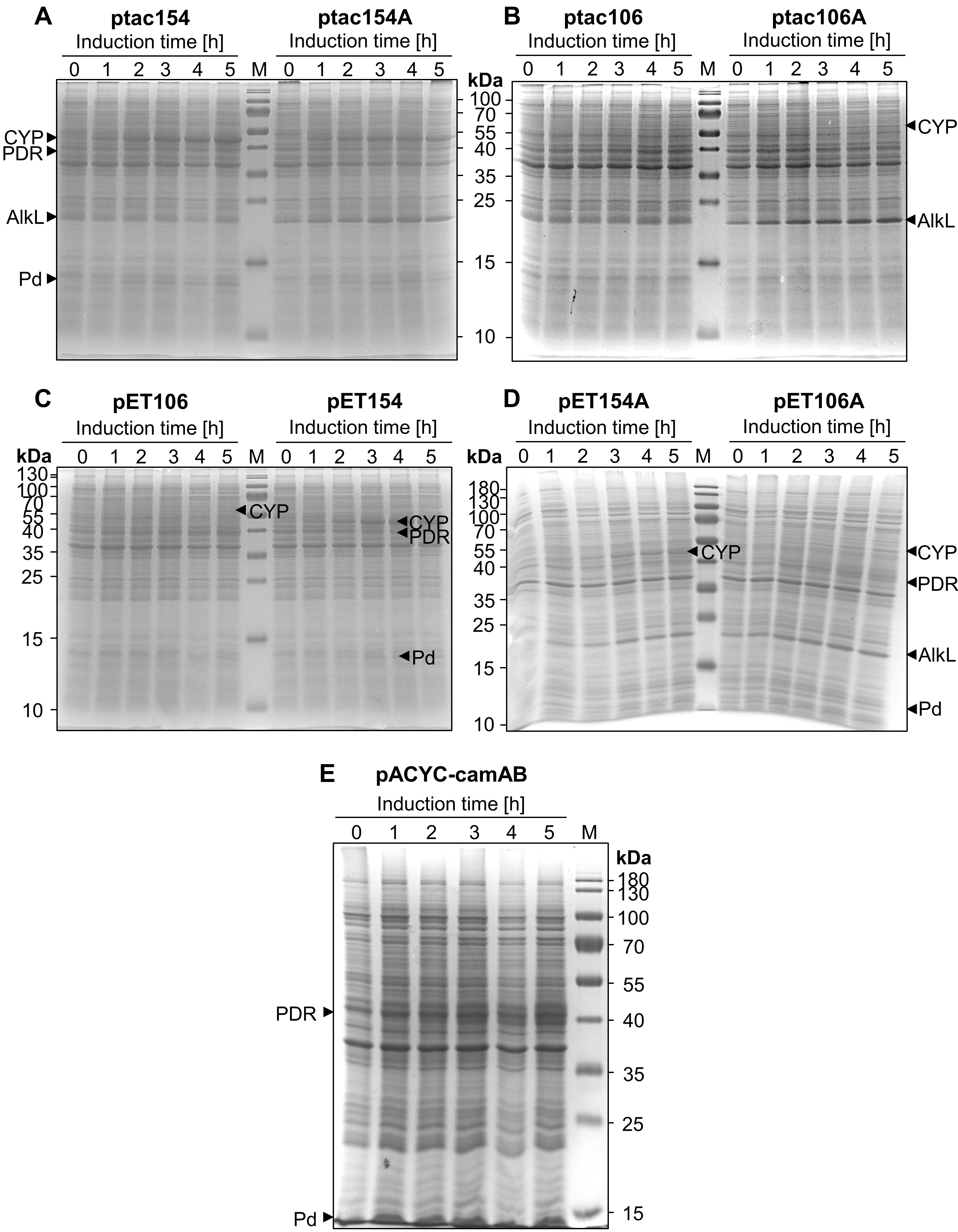

Supplement: S9 Fig — Bacteria were grown in M9 medium containing 0.5% (w/v) glucose and heterologous gene expression was induced with 0.1 mM IPTG. Expected proteins are marked with arrows in the respective gel pictures. (JPG) [file pone.0309965.s012.jpg]
