# Supplementary material for: Strategies found not to be suitable for stabilizing high steroid hydroxylation activities of CYP450 BM3-based whole-cell biocatalysts
Source: PLoS One. 2024 Sep 6;19(9):e0309965. doi: 10.1371/journal.pone.0309965 (PMC11379211; doi:10.1371/journal.pone.0309965)

Raw image for main Fig 1B, captured by Quantum ST5 and VisionCapt Software Version 16.16.0.0 (Vilber Lourmat, Collégien, France).

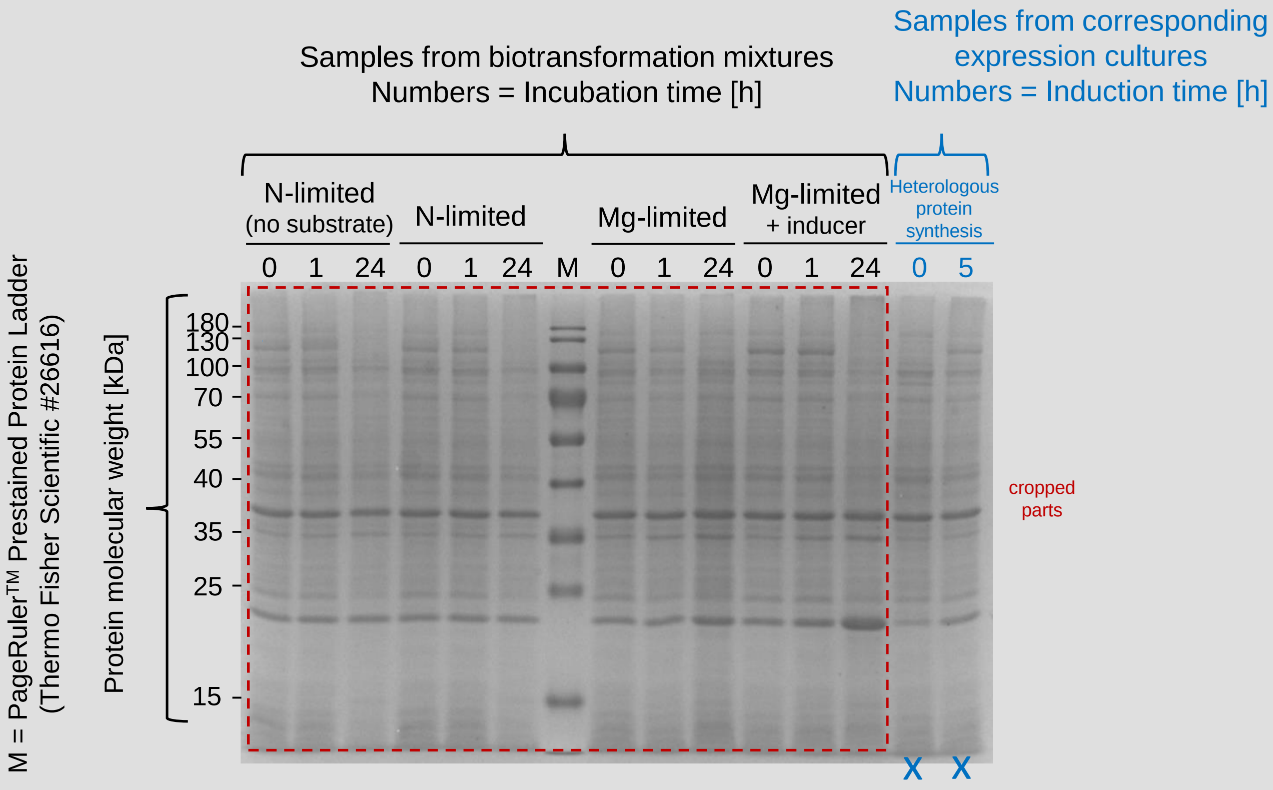

Raw images for main Fig 2B, captured by Quantum ST5 and VisionCapt Software Version 16.16.0.0 (Vilber Lourmat, Collégien, France).

Fig 2B, left:

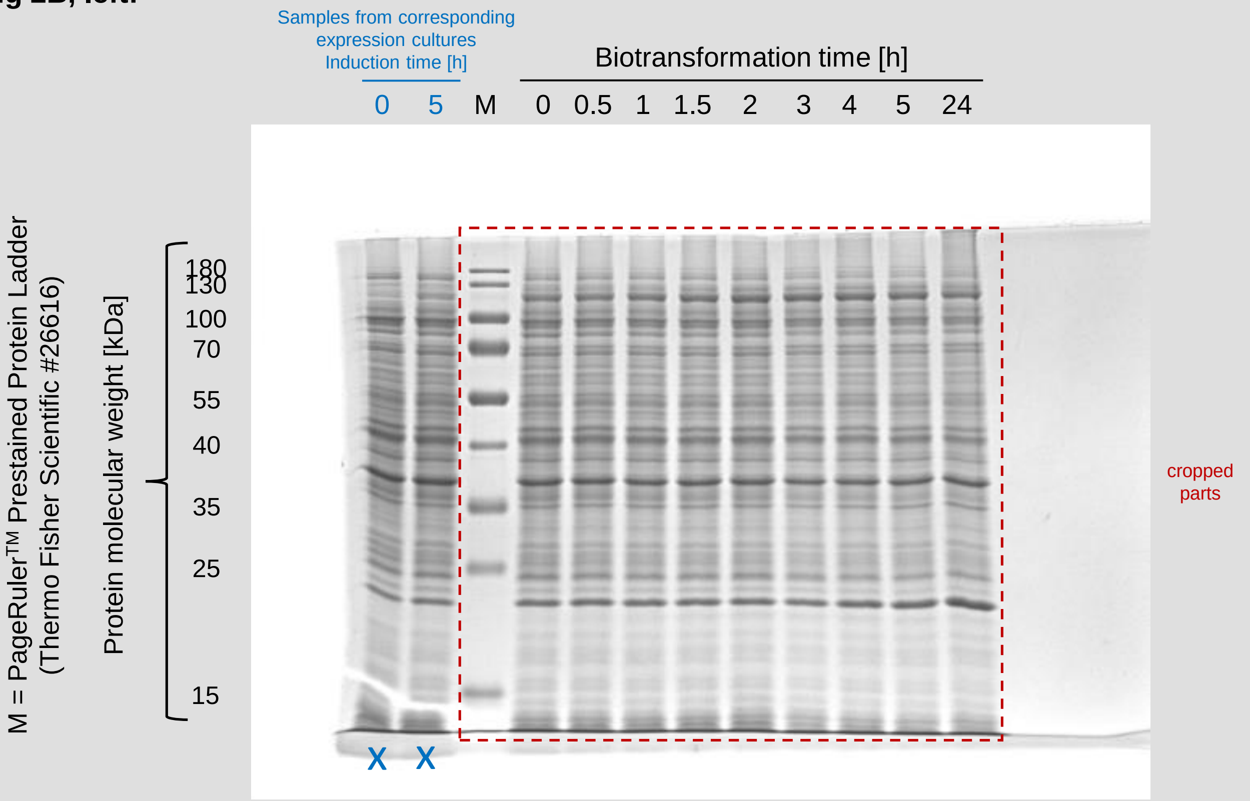

Fig 2B, right:

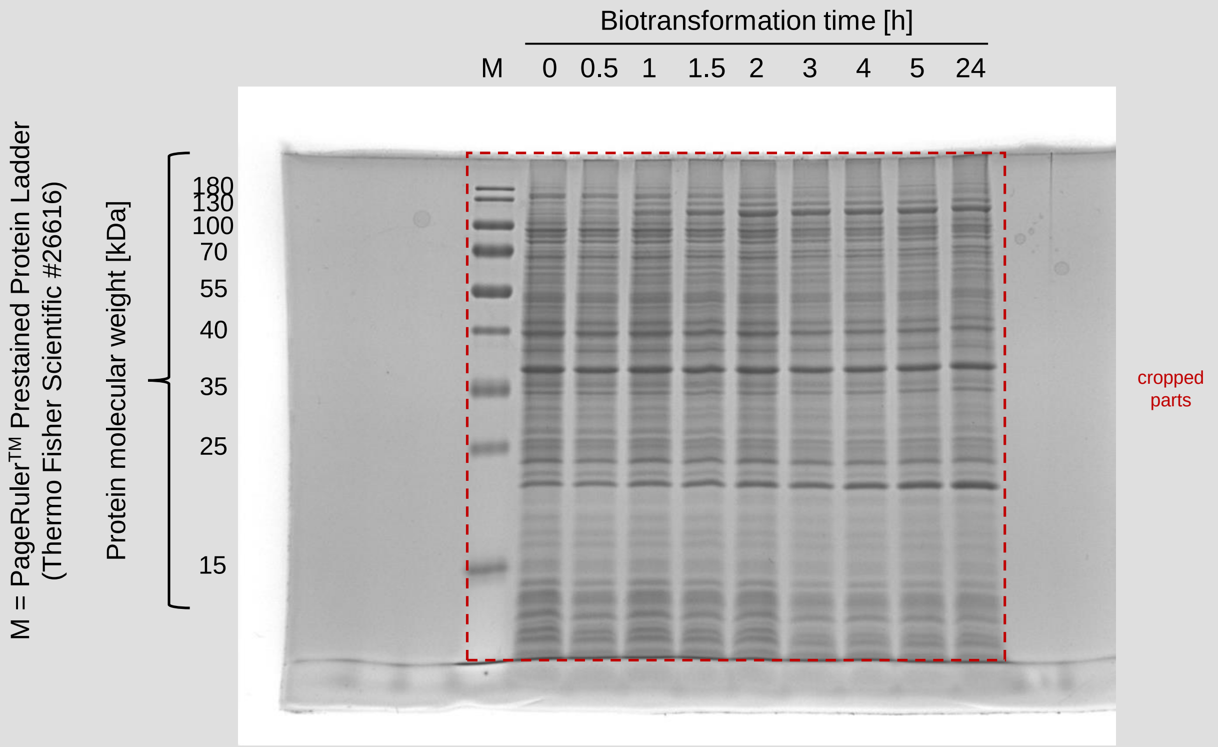

Raw image for main Fig 3B, captured by Quantum ST5 and VisionCapt Software Version 16.16.0.0 (Vilber Lourmat, Collégien, France).

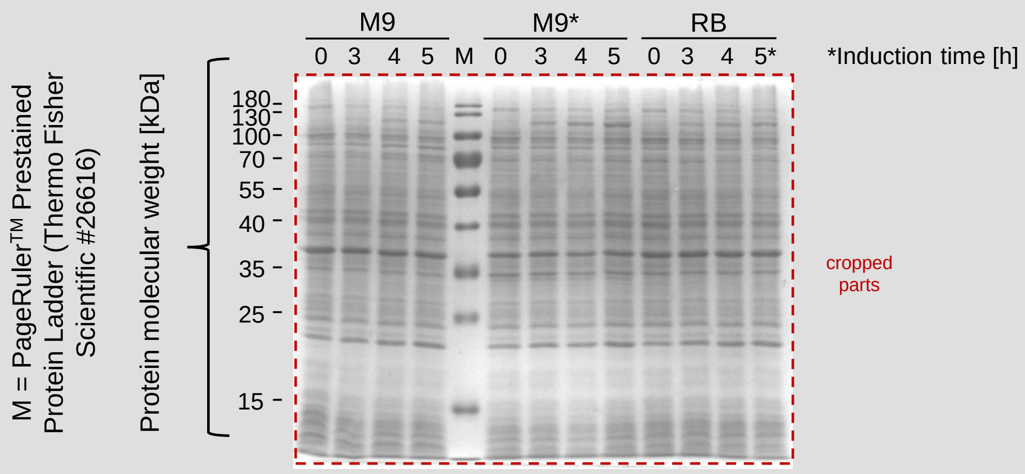

Raw image for main Fig 6B, captured by Quantum ST5 and VisionCapt Software Version 16.16.0.0 (Vilber Lourmat, Collégien, France).

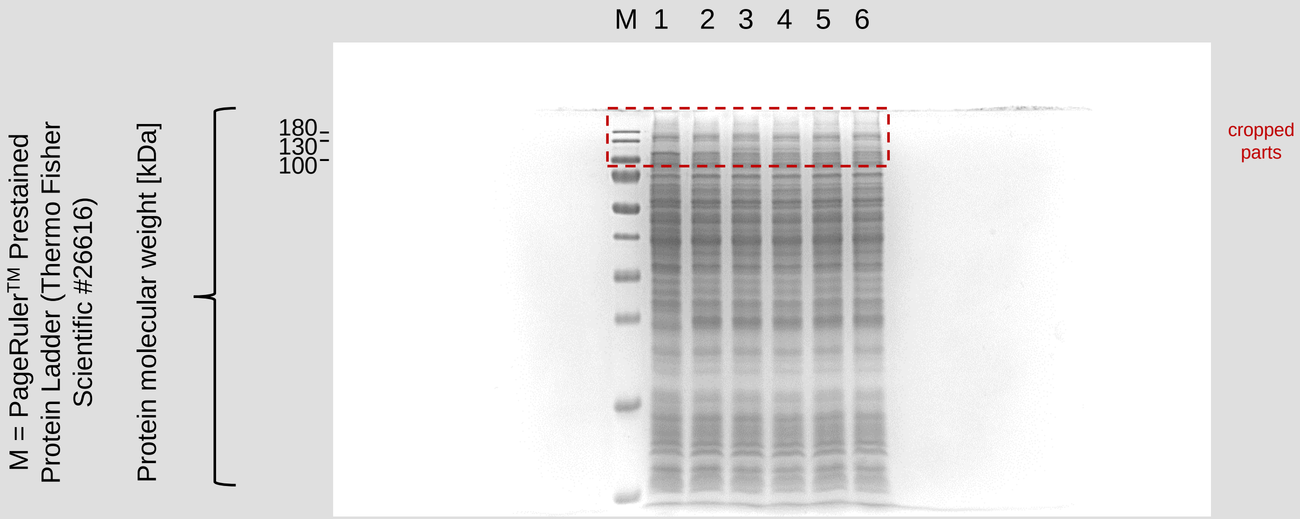

Raw images for S3A Fig, captured by Quantum ST5 and VisionCapt Software Version 16.16.0.0 (Vilber Lourmat, Collégien, France).

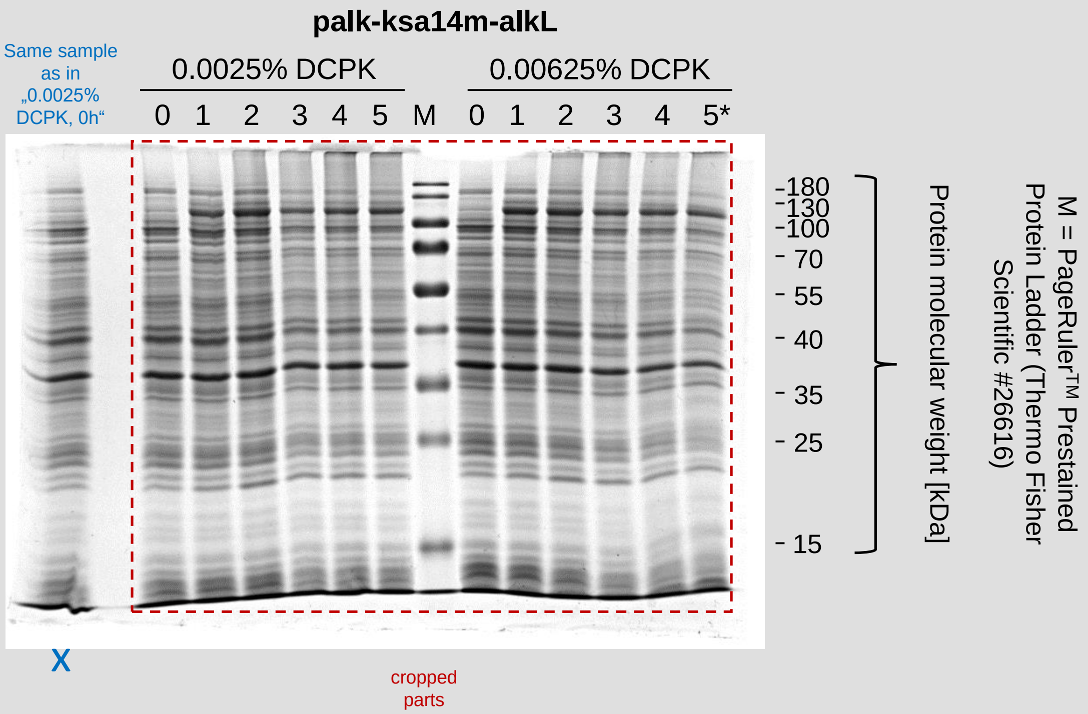

*Induction time [h]

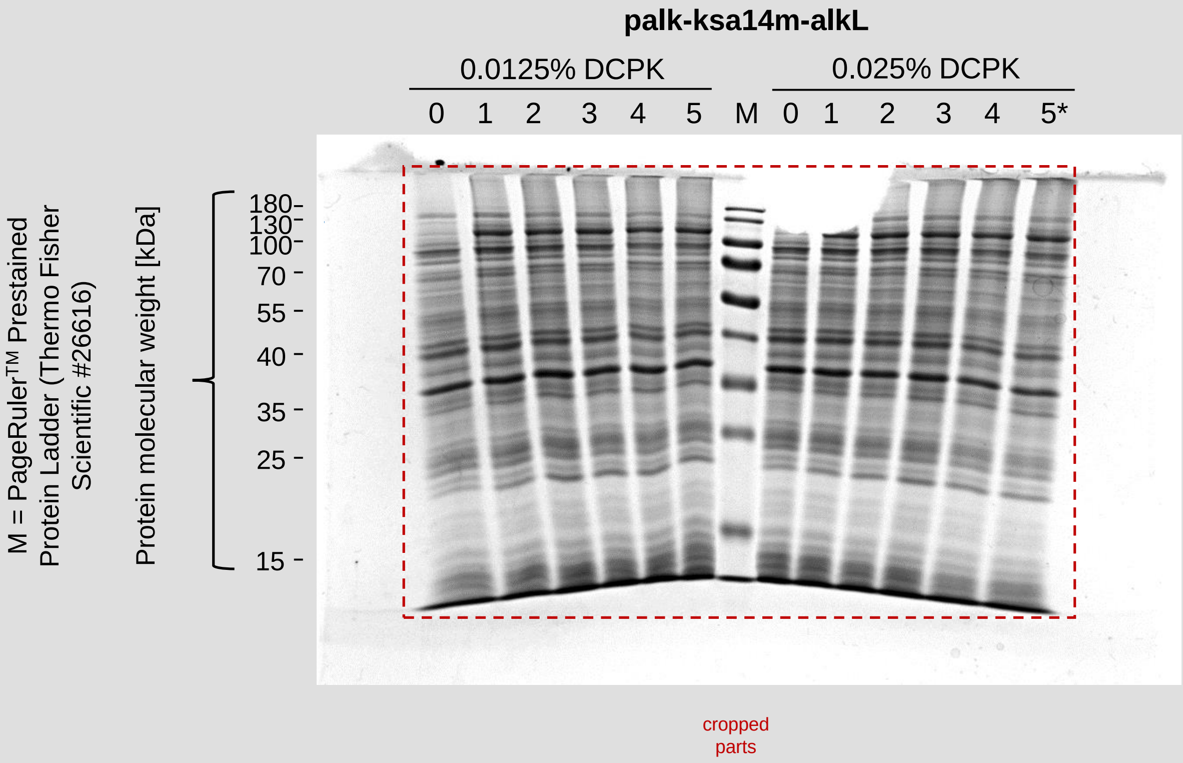

Raw image for S3B Fig, captured by Quantum ST5 and VisionCapt Software Version 16.16.0.0 (Vilber Lourmat, Collégien, France).

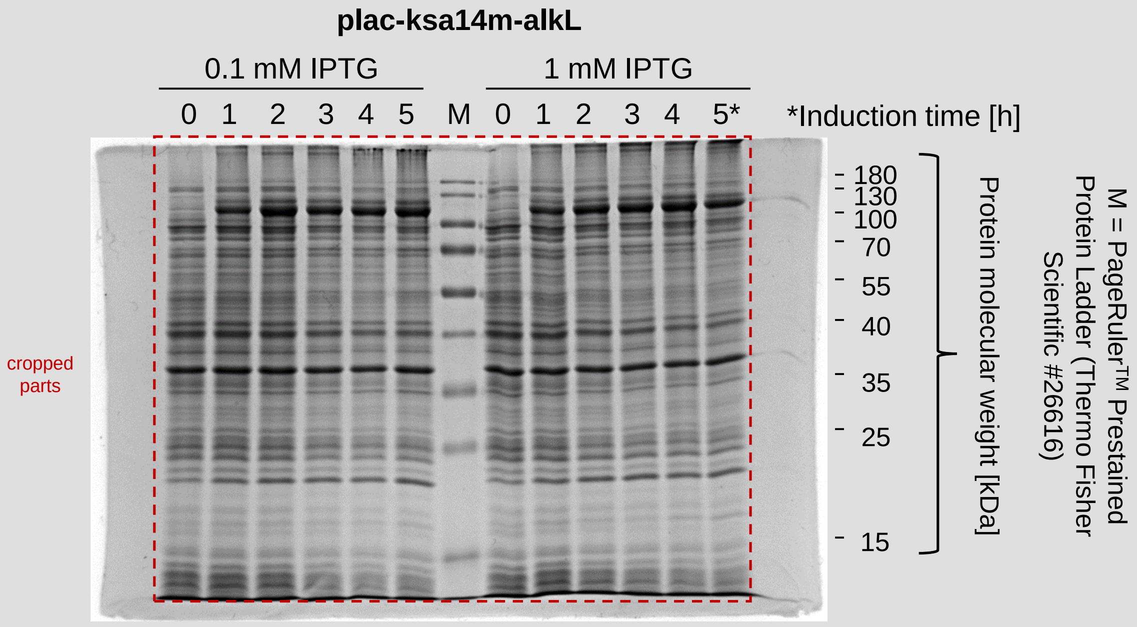

Raw image for S3C Fig, captured by Quantum ST5 and VisionCapt Software Version 16.16.0.0 (Vilber Lourmat, Collégien, France).

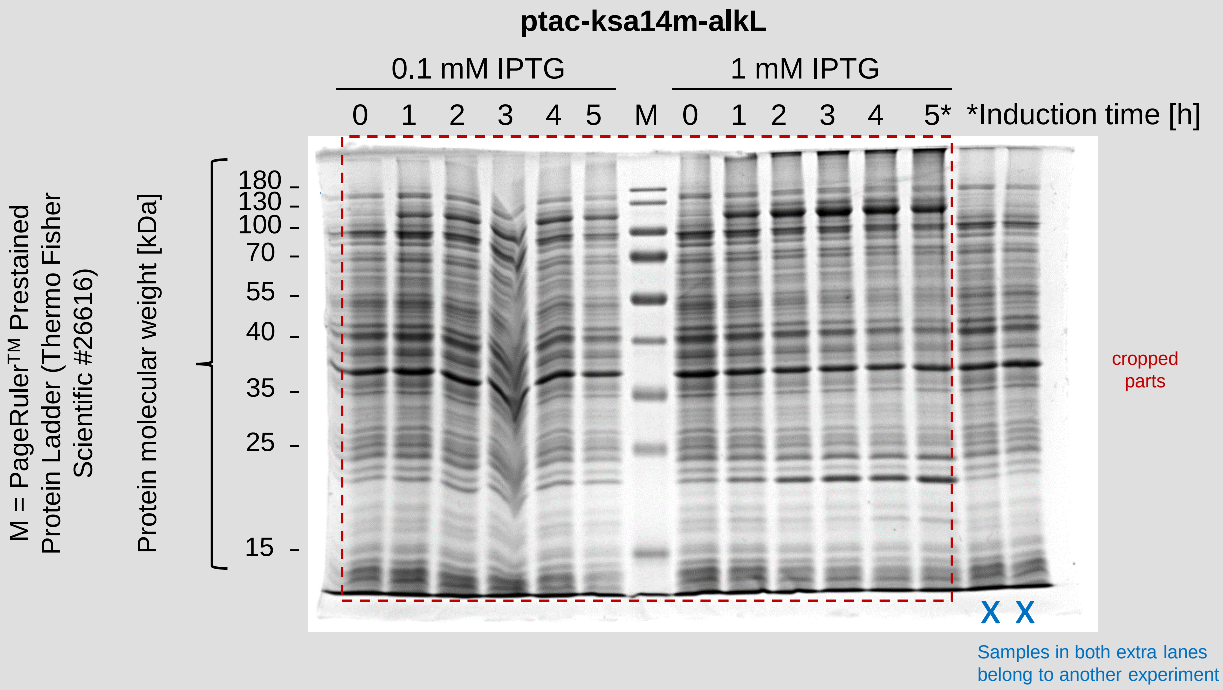

Raw image for S3D Fig, captured by Quantum ST5 and VisionCapt Software Version 16.16.0.0 (Vilber Lourmat, Collégien, France).

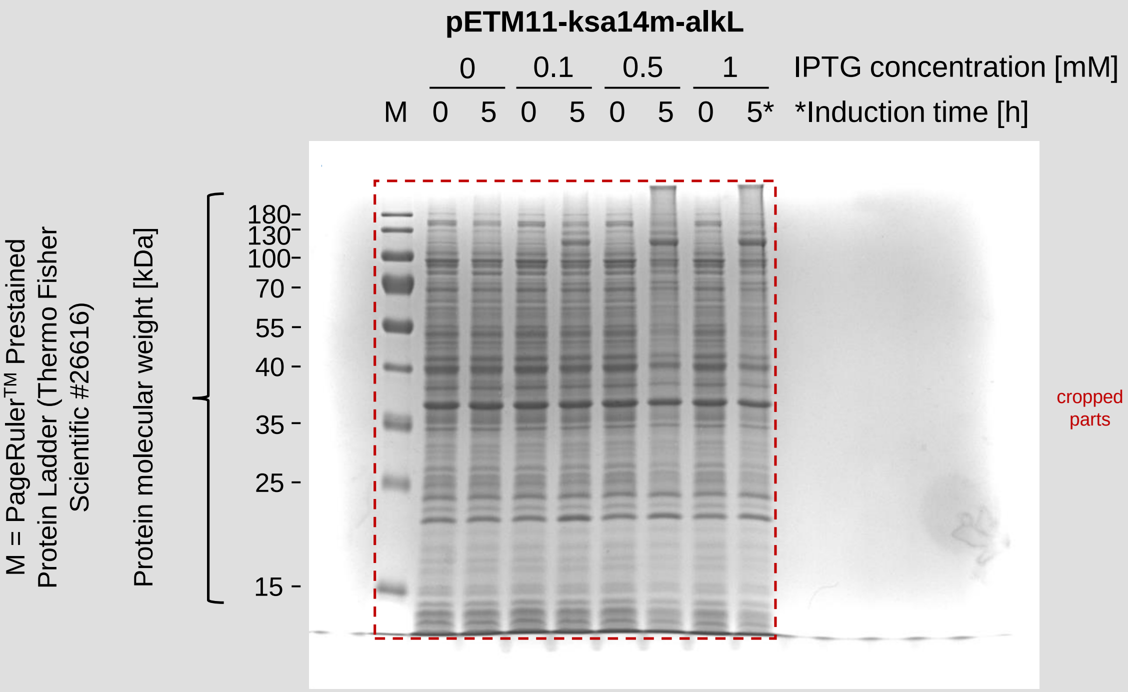

Raw image for S6B Fig, captured by Quantum ST5 and VisionCapt Software Version 16.16.0.0 (Vilber Lourmat, Collégien, France).

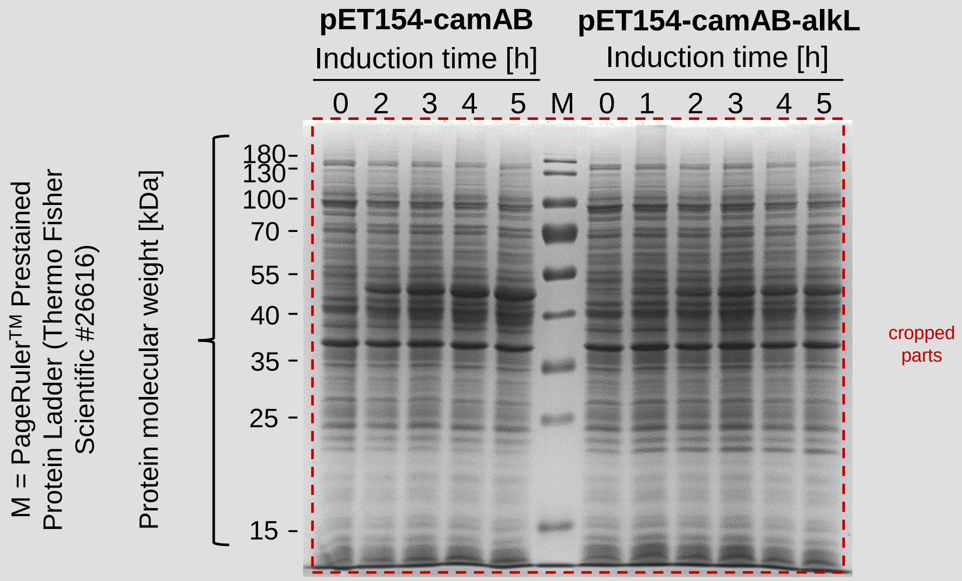

Raw image for S9A Fig, captured by Quantum ST5 and VisionCapt Software Version 16.16.0.0 (Vilber Lourmat, Collégien, France).

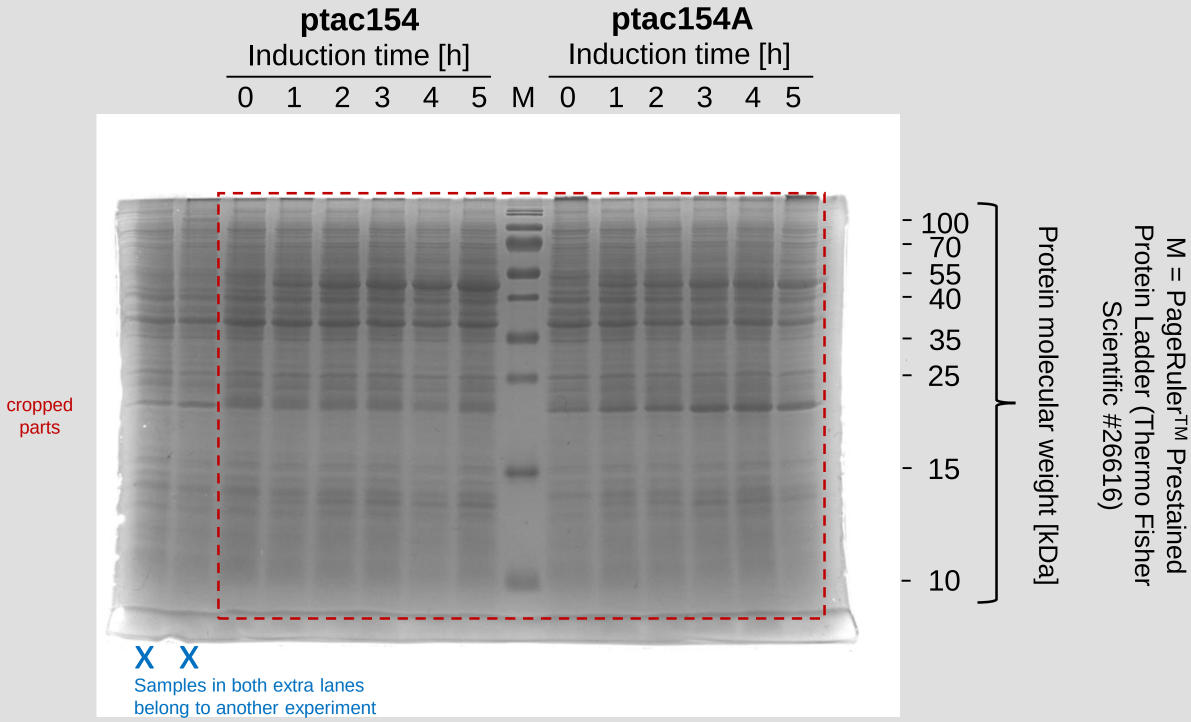

Raw image for S9B Fig, captured by Quantum ST5 and VisionCapt Software Version 16.16.0.0 (Vilber Lourmat, Collégien, France).

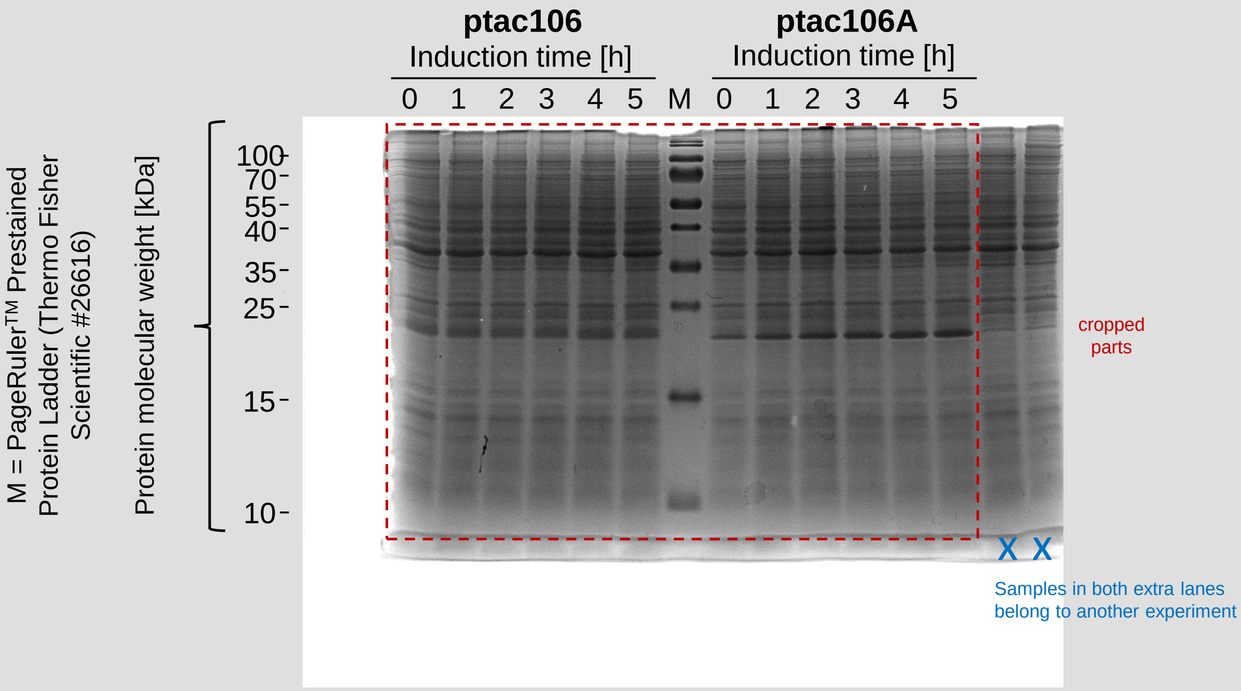

Raw image for S9C Fig, captured by Quantum ST5 and VisionCapt Software Version 16.16.0.0 (Vilber Lourmat, Collégien, France).

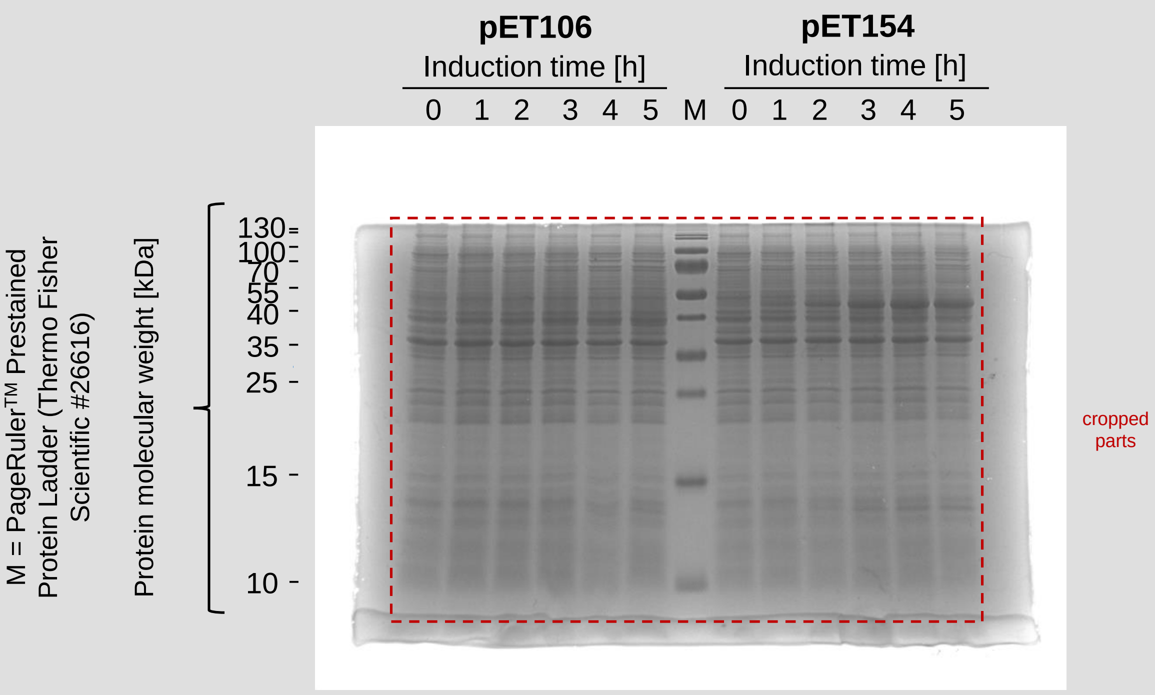

Raw image for S9D Fig, captured by Quantum ST5 and VisionCapt Software Version 16.16.0.0 (Vilber Lourmat, Collégien, France).

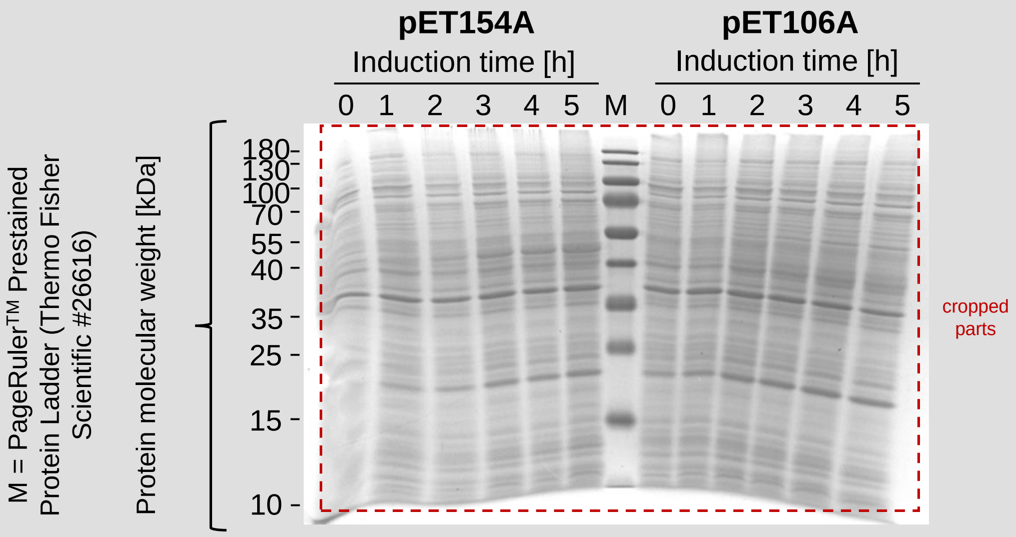

Raw image for S9E Fig, captured by Quantum ST5 and VisionCapt Software Version 16.16.0.0 (Vilber Lourmat, Collégien, France).

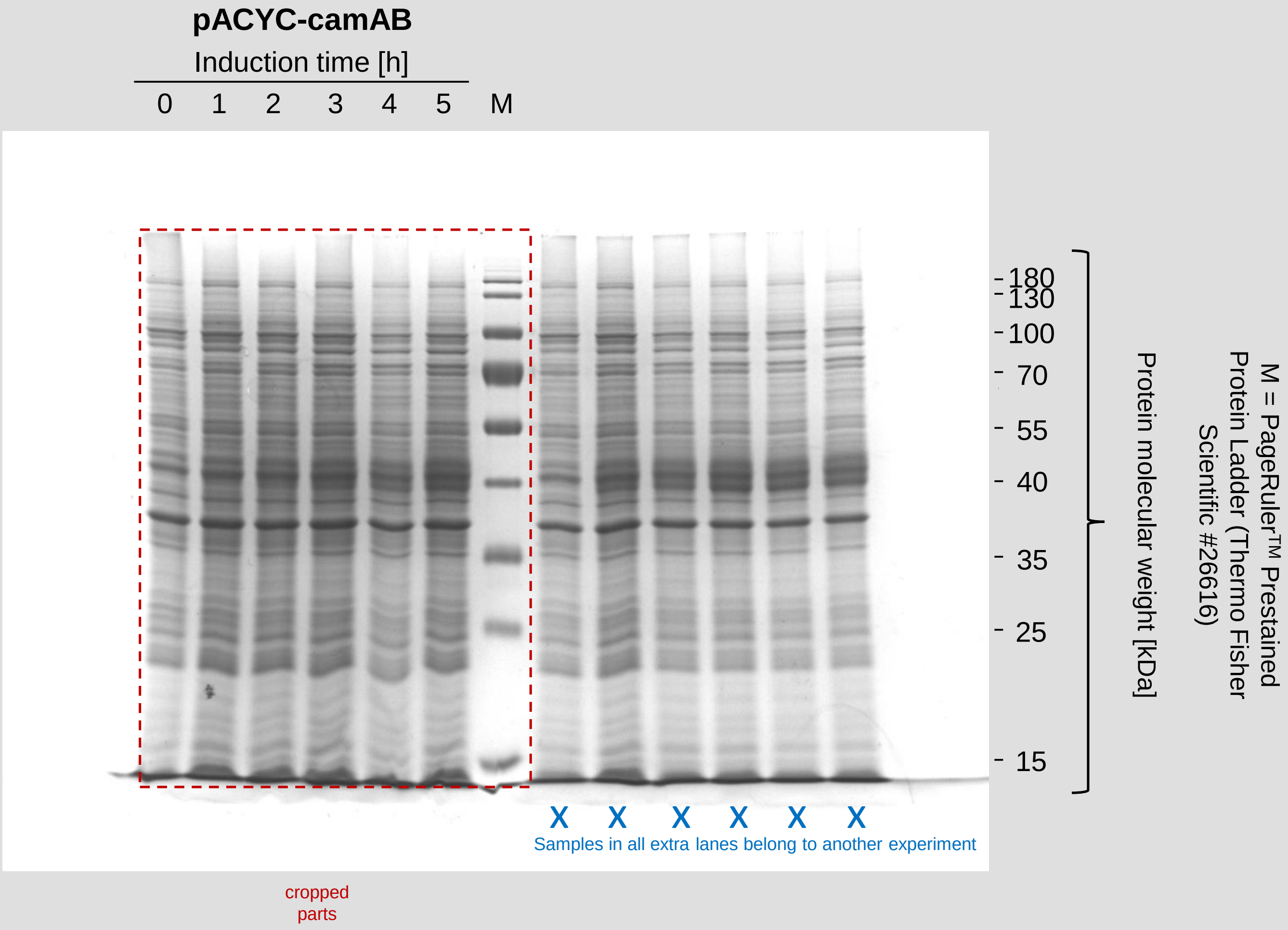

Supplement: S1 Raw images — (PDF) [file pone.0309965.s013.pdf]
