# Supplementary material for: Strategies found not to be suitable for stabilizing high steroid hydroxylation activities of CYP450 BM3-based whole-cell biocatalysts
Source: PLoS One. 2024 Sep 6;19(9):e0309965. doi: 10.1371/journal.pone.0309965 (PMC11379211; doi:10.1371/journal.pone.0309965)
Supplement: Attachment — Submitted filename: Response to Reviewers.docx [file pone.0309965.s015.docx]

**Response to editor**

Journal Requirements:

When submitting your revision, we need you to address these additional requirements.

1. Please ensure that your manuscript meets PLOS ONE's style requirements, including those for file naming. The PLOS ONE style templates can be found at 
https://journals.plos.org/plosone/s/file?id=wjVg/PLOSOne_formatting_sample_main_body.pdf and 
https://journals.plos.org/plosone/s/file?id=ba62/PLOSOne_formatting_sample_title_authors_affiliations.pdf

We have revised the manuscript and supporting data to meet the PLOS ONE style requirements according to the templates and the information given by the website.

2. Please note that funding information should not appear in any section or other areas of your manuscript. We will only publish funding information present in the Funding Statement section of the online submission form. Please remove any funding-related text from the manuscript.

We have removed funding information from the Acknowledgements section of the manuscript (line 653).

3. Thank you for stating the following financial disclosure: 
   "The study was financially supported by Bayer AG (no grant number).
Website: https://www.bayer.com
B.B. aquired the funding and CB was paid from the funding.
Bayer AG released the submitted paper for publication."

Please state what role the funders took in the study.  If the funders had no role, please state: "The funders had no role in study design, data collection and analysis, decision to publish, or preparation of the manuscript." 
If this statement is not correct you must amend it as needed. 
Please include this amended Role of Funder statement in your cover letter; we will change the online submission form on your behalf.

The funders participated in project design and approved the submission of the manuscript, but had no role in study design, data collection and analysis, or preparation of the manuscript.

4. We note that your Data Availability Statement is currently as follows: All relevant data are within the manuscript and its Supporting Information files.

Please confirm at this time whether or not your submission contains all raw data required to replicate the results of your study. Authors must share the “minimal data set” for their submission. PLOS defines the minimal data set to consist of the data required to replicate all study findings reported in the article, as well as related metadata and methods (https://journals.plos.org/plosone/s/data-availability#loc-minimal-data-set-definition).

For example, authors should submit the following data:

- The values behind the means, standard deviations and other measures reported;
- The values used to build graphs;
- The points extracted from images for analysis.

Authors do not need to submit their entire data set if only a portion of the data was used in the reported study.

If your submission does not contain these data, please either upload them as Supporting Information files or deposit them to a stable, public repository and provide us with the relevant URLs, DOIs, or accession numbers. For a list of recommended repositories, please see https://journals.plos.org/plosone/s/recommended-repositories.

If there are ethical or legal restrictions on sharing a de-identified data set, please explain them in detail (e.g., data contain potentially sensitive information, data are owned by a third-party organization, etc.) and who has imposed them (e.g., an ethics committee). Please also provide contact information for a data access committee, ethics committee, or other institutional body to which data requests may be sent. If data are owned by a third party, please indicate how others may request data access.

The minimal data set now is provided in the Supporting Information file “S2_raw_data.xlsx”.

5. We note that you have included the phrase “data not shown” in your manuscript. Unfortunately, this does not meet our data sharing requirements. PLOS does not permit references to inaccessible data. We require that authors provide all relevant data within the paper, Supporting Information files, or in an acceptable, public repository. Please add a citation to support this phrase or upload the data that corresponds with these findings to a stable repository (such as Figshare or Dryad) and provide and URLs, DOIs, or accession numbers that may be used to access these data. Or, if the data are not a core part of the research being presented in your study, we ask that you remove the phrase that refers to these data.

The first manuscript included two appearances of this phrase:

In line 539, the strain *Pseudomonas taiwanensis* VLB120_Strep containing the plasmid ptac-ksa14m-alkL did not grow in the LB preculture unlike the other *Pseudomonas* strains tested. This was a visual observation not based on measured data, but the respective comment was noted in the laboratory journal. Therefore, we have removed the phrase from that sentence.

In line 547, we have added the reference to the corresponding raw image SDS gel.

6. Please review your reference list to ensure that it is complete and correct. If you have cited papers that have been retracted, please include the rationale for doing so in the manuscript text, or remove these references and replace them with relevant current references. Any changes to the reference list should be mentioned in the rebuttal letter that accompanies your revised manuscript. If you need to cite a retracted article, indicate the article’s retracted status in the References list and also include a citation and full reference for the retraction notice.

We confirm that the reference list is complete and correct. During revision, following the reviewer recommendations, 16 references have been added to the list (listed in order of appearance):

Bernhardt R. Cytochromes P450 as versatile biocatalysts. J Biotechnol. 2006;124(1):128-145. doi: 10.1016/j.jbiotec.2006.01.026

Ortiz de Montellano PR. Cytochrome P450: structure, mechanism, and biochemistry. 3 ed. New York: Springer Verlag; 2005

Urlacher VB, Eiben S. Cytochrome P450 monooxygenases: perspectives for synthetic application. Trends Biotechnol. 2006;24(7):324-330. doi: 10.1016/j.tibtech.2006.05.002

Murdock D, Ensley BD, Serdar C, Thalen M. Construction of metabolic operons catalyzing the de novo biosynthesis of indigo in Escherichia coli. Biotechnology (N Y). 1993;11(3):381-386. doi: 10.1038/nbt0393-381

Guengerich FP. Rate-limiting steps in cytochrome P450 catalysis. Biol Chem. 2002;383(10):1553-1564. doi: 10.1515/BC.2002.175

Narhi LO, Fulco AJ. Characterization of a catalytically self-sufficient 119,000-dalton cytochrome P-450 monooxygenase induced by barbiturates in Bacillus megaterium. J Biol Chem. 1986;261(16):7160-7169. doi: 10.1016/S0021-9258(17)38369-2

Narhi LO, Fulco AJ. Identification and characterization of two functional domains in cytochrome P-450BM-3, a catalytically self-sufficient monooxygenase induced by barbiturates in Bacillus megaterium. J Biol Chem. 1987;262(14):6683-6690. doi: 10.1016/S0021-9258(18)48296-8

Tegel H, Ottosson J, Hober S. Enhancing the protein production levels in Escherichia coli with a strong promoter. FEBS J. 2011;278(5):729-739. doi: 10.1111/j.1742-4658.2010.07991.x

Terpe K. Overview of bacterial expression systems for heterologous protein production: from molecular and biochemical fundamentals to commercial systems. Appl Microbiol Biotechnol. 2006;72(2):211-222. doi: 10.1007/s00253-006-0465-8

Makart S, Heinemann M, Panke S. Characterization of the AlkS/PalkB-expression system as an efficient tool for the production of recombinant proteins in Escherichia coli fed-batch fermentations. Biotechnol Bioeng. 2007;96(2):326-336. doi: 10.1002/bit.21117

Panke S, Wubbolts MG, Schmid A, Witholt B. Production of enantiopure styrene oxide by recombinant Escherichia coli synthesizing a two-component styrene monooxygenase. Biotechnol Bioeng. 2000;69(1):91-100. doi: 10.1002/(sici)1097-0290(20000705)69:1<91::aid-bit11>3.0.co;2-x

Wang Y, Rawlings M, Gibson DT, Labbé D, Bergeron H, Brousseau R, et al. Identification of a membrane protein and a truncated LysR-type regulator associated with the toluene degradation pathway in Pseudomonas putida F1. Mol Gen Genet. 1995;246(5):570-579. doi: 10.1007/BF00298963

Ruff AJ, Arlt M, van Ohlen M, Kardashliev T, Konarzycka-Bessler M, Bocola M, et al. An engineered outer membrane pore enables an efficient oxygenation of aromatics and terpenes. J Mol Catal B Enzym. 2016;134:285-294. doi: 10.1016/j.molcatb.2016.11.007

Lee MJ, Kim H-J, Lee J-Y, Kwon AS, Jun SY, Kang SH, et al. Effect of gene amplifications in porphyrin pathway on heme biosynthesis in a recombinant Escherichia coli. J Microbiol Biotechnol. 2013;23(5):668-673. doi: 10.4014/jmb.1302.02022

Ge J, Wang X, Bai Y, Wang Y, Wang Y, Tu T, et al. Engineering Escherichia coli for efficient assembly of heme proteins. Microb Cell Fact. 2023;22:59. doi: 10.1186/s12934-023-02067-5

Hu B, Yu H, Zhou J, Li J, Chen J, Du G, et al. Whole-cell P450 biocatalysis using engineered Escherichia coli with fine-tuned heme biosynthesis. Adv Sci. 2023;10(6):e2205580. doi: 10.1002/advs.202205580

Additional Editor Comments:

Dear Bruno

you will see the comments from the reviewers are supportive and I am looking forward to read the revision. And, as it was hard to appoint reviewers in time and thus we reached holiday saison I understand that it may take time. Still I hope you will improve and send a next version to make a decision.

Sincerely
Dirk

**Response to reviewers**

Reviewer #1: In their manuscript “Strategies found not to be suitable for stabilizing high steroid hydroxylation activities of CYP450 BM3-based whole-cell biocatalysts”, Bertelmann and Bühler explore various strategies aimed to increase the stability of whole-cell steroid-hydroxylation by Escherichia coli equipped with heterologous cytochrome P450 monooxygenases (CYP450s). Approaches include biotransformation media/buffers with different nutrient compositions, growing cells vs. resting cells, different growth media, different expression systems (prompters, inducers, inducer concentrations), protein engineering, using Pseudomonas as a host, and testing different CYP450 enzymes.

In general, although the authors state that they did not achieve a better system (but see my comment on that below), the work seems to be carefully conducted and is of interest for applied studies.

However, the manuscript should be presented in a more reader-friendly way. The authors assume a lot of knowledge in the abstract and the introduction (see comments).
Also, although the results might indeed be a guideline for further studies, the article is scientifically somewhat unsatisfying, because likely reasons for decreasing activity are discussed, but no data are shown. For example, what about levels of O2 in the biotransforming cell cultures, heme content in CYP450s, or NAD(P)H:NAD(P)+ ratios? If there are values known from literature, I suggest mentioning them, perhaps in the introduction (see my comment Nr. 2).

Finally, I do not understand why the authors did not proceed with, and did not do more experiments on, the plac system (Fig. 4B), and particularly the 0.1 mM IPTG / 0.1 g CDW setup (see my comment Nr. 6). This should be commented on.

In summary, in my opinion, the article could be published without further experiments, but with a thorough editing of the text.

Comments in detail:
1. The abstract is a bit confusing for readers not familiar with this field and the preceding study, respectively. Perhaps the authors may shorten the description of results to make some room for briefly summarizing the former setup that they aimed to optimize.

We have revised the abstract according to the reviewer’s suggestion (lines 18-33, 40).

2. The same holds true for the introduction. It would benefit from introducing CYP450s a bit, such as specifying their cofactors and redox partners, or what is meant by “multi-component nature” (line 48). Also, the previous system should be introduced better, e.g., what is meant by “self-sufficient” (line 51), what is uncoupling (line 62; although this is explained later), what is the KSA14m variant (line 64)… It would also help the readers a lot if you introduced possible optimization steps (or bottlenecks) before delving into the results.

We agree with this comment and thus have revised the introduction as recommended (lines 44-45, 47-60, 64-71, 74-81, 89, 92). The purpose of this study is now mentioned more clearly in the introduction (lines 84-86) as well as in the beginning of the Results&Discussion section (lines 240-242). Possible optimization steps/bottlenecks are introduced in lines 98-108.

3. Lines 167-168: When the cells are resuspended in potassium phosphate buffer, they are depleted for every nutrient (except K, P; and glucose). Could this influence catalysis and/or stability? For example, what happens with a CYP450 when iron becomes limiting? Why did you not use M9 without nitrogen?

The lack or depletion of nutrients can indeed affect catalysis as well as stability due to their respective demand in the whole-cell biocatalyst’s metabolism. In this study, the standard biotransformation setup with resting (i.e., non-growing but metabolically active) cells included potassium, phosphate, and glucose as sole components, as it also has broadly been reported in literature. The lack of a nitrogen source thereby is used to achieve a growth limitation, which transfers the cells into a resting, but metabolically active state. In this state, competition of metabolic demands of the biocatalytic reaction with cellular maintenance and biomass formation is reduced. This often allows superior specific biotransformation rates of resting cells compared to growing cells (as also observed in this study, Figs. 1 and 2), ultimately resulting in increased product yields on energy source as well as facilitated reusability and downstream processing. However, omitting nitrogen from the biotransformation medium implies a down-regulation of biosynthetic capacities, especially the efficient (re‑)synthesis of enzymes and energy/redox carriers, which can affect the operational stability during the biotransformation (explained in lines 251-263, 277-283). This is the reason why we tested M9 without magnesium to exclude effects of other nutrients including nitrogen or iron. Similar activities were observed. Just deprivation of magnesium still allows some growth as shown in an earlier study, in which M9 medium without magnesium allowed better performance than M9 medium without nitrogen, whereas the latter did not result in differences compared to Kpi buffer with glucose (Willrodt C, Hoschek A, Bühler B, Schmid A, Julsing MK. Decoupling production from growth by magnesium sulfate limitation boosts de novo limonene production. Biotechnol Bioeng. 2016;113(6):1305-1314. doi: 10.1002/bit.25883). This information was added (lines 263-267).

4. Results: Specific activity does not correlate with the enzyme level (e.g., Fig. 1A; Fig. 2). Have you ever purified the enzyme from the cells and checked its cofactor load? Or else, could oxygen be a limiting factor? Since you add glucose, I suppose that the cells have a high respiration rate? The results obtained with low cell concentrations you present in Fig. 4 (and accompanying text) suggest that O2 might not be the limiting factor, but did you ever measure O2 levels in your biotransformation cultures? If you analyzed these things previously, it would be good to mention that. If not, I do not request these analyses for publication of this manuscript, but they might be worth a try in the future.

We initially aimed to purify the enzyme based on the 6x-His-tag at the N-terminal end of the enzyme for a thorough evaluation of its specific kinetic properties and uncoupling behavior (i.e., uncoupling of NADPH oxidation and substrate hydroxylation). Our attempts however were not successful due to impurity of the final eluates, which may severely influence the respective experimental outcome.

Regarding the measurement of oxygen in the biotransformation cultures: Oxygen levels were not measured for the experiments presented in this manuscript and therefore no data is available in this regard. We agree that oxygen-dependent reactions in whole-cell biocatalysis can be limited by oxygen mass transfer, especially in closed systems. In our study, assays featured a volume ratio of liquid : headspace of 1 : 11, and, for reaction time course analysis, tubes were opened regularly for sampling. Moreover, in our previous study, we investigated oxygen as limiting factor by varying biomass concentrations in resting-cell biotransformation setups (Bertelmann C, Mock M, Schmid A, Bühler B. Efficiency aspects of regioselective testosterone hydroxylation with highly active CYP450-based whole-cell biocatalysts. Microb Biotechnol. 2024;17(1):e14378. doi: 10.1111/1751-7915.14378). Initial activities only were hampered by oxygen mass transfer at the highest cell concentration tested (1.89 g_CDW_ L^-1^), indicating that the system was not oxygen mass transfer limited at a cell concentration of 1 g_CDW_ L^-1^ (which is the maximum cell concentration applied in this study).

This study’s experiments were conducted in small scale (1 mL liquid volume), to evaluate strategies typically applied for oxygenase stabilization, and served as preliminary considerations for follow-up experiments in bioreactors under real process conditions. Scale-up of the investigated biotransformation would involve increased biomass concentrations, accompanied by an increased demand for oxygen. Such experiments are ongoing featuring a thorough monitoring of process parameters such as pO_2_ as well as its continuous supply.

5. Depending on your response to 4., it might also be worth mentioning that E. coli strains with optimized heme uptake or production have been generated.

Thank you for this recommendation. We have added respective information and references in lines 550-552.

6. Also results, Fig. 4: why did you not follow up on the plac system (Fig. 4B), and the 0.1 mM IPTG / 0.1 g CDW setup in particular? It seems to me that it stabilized, although on a somewhat lower than maximally achievable level. Still, did you ever measure activity at later time points? Did you quantify product yields?

The strain carrying plac-ksa14m-alkL indeed exhibited an improved operational stability in the 0.1 mM IPTG / 0.1 g_CDW_ L^‑1^ setup. Such low cell concentration however involves very low space-time yields, excluding further development of technical applications. Additionally, our previous study identified a limited availability of the biotransformation substrate testosterone as well as product inhibition as further challenges for KSA14m-catalyzed testosterone hydroxylation. The stabilization of specific activity thus likely results from the increased testosterone availability and lower product formation at low cell concentrations, which is corroborated by the fast activity decrease at higher cell concentrations (lines 450-455).

7. Do the results shown in Fig. 5B not argue for enzyme destabilization independent from substrate / product concentrations? This could be mentioned much earlier in the manuscript to avoid questions piling up.

This statement has been added to the introduction (lines 75-77) as well as respective results session (line 499-500) with a reference to our previous publication.

8. Lines 495-496: It would be nice for the reader if you would explain what fhuA and todX mean. Also, I assume you want to refer to Fig. 6, not 4.

TodX and FhuA Δ1-160 are outer membrane proteins which have been shown to facilitate testosterone uptake into *E. coli* in our previous study (Bertelmann C, Mock M, Schmid A, Bühler B. Efficiency aspects of regioselective testosterone hydroxylation with highly active CYP450-based whole-cell biocatalysts. Microb Biotechnol. 2024;17(1):e14378). We have added this information and their respective origin to the manuscript (lines 532-537). The reference has been changed to Fig. 6 (line 538), you are correct.

9. Fig. 6 and results with Pseudomonas, respectively: Obviously, CYP350 levels are not very high in this strain. Did you consider changing expression vectors before dismissing this species completely?

The chosen expression systems have proven highly suitable for the heterologous synthesis of other oxygenases in several *Pseudomonas* strains including VLB120, enabling good expression and high specific activities (line 530-532). In this study, applied systems did not lead to a successful outcome. We agree that using other expression vectors may allow for a better performance of resulting *Pseudomonas* strains with regard to growth, heterologous protein synthesis, catalytic activity, and stability. For instance, a significant improvement of CYP450-catalyzed cyclohexane hydroxylation using *Pseudomonas taiwanensis* VLB12_Strep as host organism has been achieved *via* systematic genetic engineering (Schäfer L, Karande R, Bühler B. Maximizing biocatalytic cyclohexane hydroxylation by modulating cytochrome P450 monooxygenase expression in P. taiwanensis VLB120. Front Bioeng Biotechnol. 2020;8:140). A respective comment has been added in lines 552-555). Further work on this topic however was out of the scope of this study.

10. Line 545: To evaluate this system, it is important to know the putidaredoxin levels. At least for selected conditions, this should be done, e.g. by higher-percentage gels and/or a Tris/Tricine gel.

This section briefly demonstrates differences in the performance of whole-cell systems equipped with single-component CYP450 BM3 variants or native, multi-component CYP450 systems by using experimental standard setups. We agree that optimizing the SDS-PAGE setup could enable the visualization of putidaredoxin levels and thus more thorough evaluation of the system, which however was out of the scope of this study.

11. Conclusions: The manuscript is somewhat overwhelming. I suggest showing a simplified summarizing table.

We have added Table 3 to the manuscript as suggested (lines 647-649).


Minor:
12. Fig. 2A, left panel: the product symbols look like the testosterone symbols after 1 h.

We have adjusted the symbol shape in Figs. 2A and B for better differentiation between testosterone and product symbols.

13. Results, Fig. 4 and Fig. 5: Please indicate which resting cell system was applied.

Respective information has been added in line 383 for Fig. 3, line 438-439 for Fig. 4, and line 507-508 for Fig. 5.

14. Line 440 and line 446: Is it position 87 or 82? Please specify.

In line 440ff (in the revised manuscript: line 467-471), position 87 is described as key site for mutation fostering uncoupling. However, this mutation is required for CYP450 BM3 to accept testosterone as substrate in the first place. This is why KSA14m was not further engineered at this position. Alternatively, mutation of position 82 (which has been done to obtain KSA14m) has been identified to enhance uncoupling (line 446 in the previous manuscript). We thus chose to reintroduce the original alanine at this position by generating the variant KSA14m_M82A. Information has been added in line 480-483 for better comprehension.

15. Table 2 is hard to understand; I’d draw lines between the rows in the lower part.

We have improved Table 2 and its caption for easier understanding (lines 627-631).

Reviewer #2: Previously, the same group investigated a whole-cell E. coli-based biocatalyst co-expressing the outer membrane pore AlkL and the P450 BM3 variant KSA14m capable of hydroxylating testosterone primarily to the 15-b-hydroxylated product. During biotransformation, resting cells lose activity due to instability of BM3 monooxygenase. In the present study, the authors tested a number of approaches and varied several factors in an attempt to increase enzyme stability during the process, which could increase the duration of biotransformation and thus lead to higher overall product concentrations. Among other things, they compared resting and growing E. coli cells, varied nutritional conditions during gene expression, different media, promoters, and inducer concentrations. None of these changes resulted in improved cell activity or increased stability of BM3 KSA14m during biotransformation. Interestingly, culture medium and expression strategies influence the initial whole cell activity but not its stability. Furthermore, two additional stabilizing mutations were introduced into the monooxygenase gene, which ultimately did not change the product titer. The use of Pseudomonas instead of E. coli did not improve productivity. Two other steroid hydroxylating P450s, CYP154C5 and CYP106A2, were cloned in E. coli together with the two redox partner genes. While CYP106A2 could not be expressed at all, the biotransformation of testosterone by cells expressing CYP154C5 was found to be no more productive than cells with BM3 KSA14m.

In general, although no improvement could be achieved within this study, the "negative" results can be very helpful for further studies on optimization of whole cell biocatalysts for oxygenation reaction.
I recommend to publish the paper after some revisions:
1. As shown in Figure 2, growing cells continued to express KSA14m even after 24 h of biotransformation (compared to resting cells), but this did not lead to higher biocatalyst productivity. This was explained by a stronger competition of enzyme synthesis and metabolic demands of the reaction with the demands of cellular maintenance and biomass formation. It seems that cell activity (independently if growing or resting) is dependent on the P450 concentration per 1 g cww, as clearly shown in Fig. 2a and Fig. 2b. In the case when the induction was done simultaneously with substrate addion, the specific cell activity first increases with increasing P450 concentration (see the corresponding SDS gel) and then decreases with complete loss after 3.5 h. It would be interesting to measure P450 concentration (using CO difference spectrum). It will not change the situation with the loss of activity after a certain time of biotransformation, but it would allow a more concrete explanation that specific cell activities in growing cells were lower (even at the very beginning of the biotransformation) compared to the resting cells due to lower P450 concentration per cww.

We agree that measuring the active CYP450 concentration per gram of cell dry weight would allow a superior view on the activity loss, as SDS-PAGE analyses indeed do not give information on the active portion of an enzyme. CO difference spectra usually constitute the method of choice for such quantification. Respective methodology (well-established for other CYP450s employed in our working group) did however not give absorption peaks for the described CYP450 BM3 variant KSA14m, neither at 450 nor at 420 nm.

2. It would be helpful for the readers if the genetic construct- AlkL and KSA14m expressed from the same operon – will be described in the introduction section.

Respective information has been added in lines 64-67.

3. L. 316-317: “Obviously, KSA14m experienced a similar inactivation in growing as in resting cells,which could not be alleviated by enzyme resynthesis in growing cells”. Compared to resting cells in which the P450 band disappeared after 24 biotransformation, the band corresponding to the P450 remains in growing cells. Could it be that the loss of activity is not only due to protein instability, but due to the loss of heme or FAD or FMN (the prosthetic groups of the reductase domain of P450 BM3)? None of these aspects have been checked in this study, but it is importnat to at least mention them in the discussion.

Thank you for this suggestion, which we have integrated into the manuscript (lines 339-341).

4. L. 375-377: Please mention the promoters used in this study and their strength compared to each other to make clearer their choice for this study.

The explanation has been added in lines 402-406.

5. Was CYP106A2 not active in whole cells or was cloning unsuccessful?

In the first approach (one plasmid strategy), cloning of the *cyp106a2* gene was not successful (information has been added to line 584). In the second approach (two plasmid strategy), cloning of the *cyp106a2* gene was achieved (see line 610-612). The resulting strains however did not or only barely display testosterone hydroxylation activity (Table 2).

6. Fig.2 In both cases, 700 mkM product concertation was achieved after 2.5 – 4 h starting with 1 mM (=1000 mkM) testosterone. Was the productivity of resting cells used at the same cell density higher?

The productivity (space-time yield) of resting cells was indeed higher than that achieved with growing cells at the same cell density and initial testosterone concentration after the same induction time. While 700 µM product were measured after 2.5-4 h with growing cells, resting cells reached a concentration of 800 µM already after 1 h of biotransformation (we have added S2 Fig to the Supporting Material, caption see lines 959-964 in the revised manuscript). This statement as well as corresponding space-time yields are given in the revised manuscript (lines 343-346).

Reviewer #3: The manuscript presents negative results, however of important biotechnology field. The authors performed many rigorous, well documented and analyzed experiments but were not able to reach desired stability of whole cell oxidation of steroids.
I have only a few recomendations:
1. I am missing a discussion which would discuss more widely the impacts of the findings both to the scientific field as well as to the biotechnology praxis.

We have broadly revised the abstract and introduction to clarify the relation to the scientific field and explain the current biotechnology praxis (lines 18-29, 47-83). We consider this study as a collection of valuable approaches and their effects for the stabilization of whole-cell biocatalysis – not only for the investigated CYP450 BM3 variant KSA14m, but for oxygenases in general (lines 35-36, 639-642). The great potential and value to follow up further optimization of this specific biocatalyst/biotransformation is shown by the comparison with alternative steroid-hydroxylating CYP450s, which revealed its superior specific activity and thus higher productivity (lines 36-38, 625-626, 642-644). One can further conclude from this study that enzyme engineering *via* rational or random mutagenesis represents a promising strategy to stabilize testosterone hydroxylation by the described whole-cell biocatalysts (lines 38-40, 516-519, 642-644). The references list has been amended accordingly (see above).

2. English is of high level, except of a few wrong articles:
- "the" missing in front of the "most" on several places.

This rule applies only situationally and respective changes done do not include all appearances of “most”. We leave further possible changes to the editorial finishing.

- "a first approach" should be "the" (line 535).

We changed this accordingly (line 582).

While revising your submission, please upload your figure files to the Preflight Analysis and Conversion Engine (PACE) digital diagnostic tool, <https://pacev2.apexcovantage.com/>. PACE helps ensure that figures meet PLOS requirements. To use PACE, you must first register as a user. Registration is free. Then, login and navigate to the UPLOAD tab, where you will find detailed instructions on how to use the tool. If you encounter any issues or have any questions when using PACE, please email PLOS at [figures@plos.org](mailto:figures@plos.org). Please note that Supporting Information files do not need this step.

We uploaded all figure files to the PACE tool, which confirmed that Figs. 1-7 meet PLOS requirements (see screenshot attached):
